# TCF-1 regulates NKG2D expression on CD8 T cells during anti-tumor responses

**DOI:** 10.1007/s00262-022-03323-0

**Published:** 2022-12-23

**Authors:** Rebecca Harris, Mahinbanu Mammadli, Shannon Hiner, Liye Suo, Qi Yang, Jyoti Misra Sen, Mobin Karimi

**Affiliations:** 1 Department of Microbiology and Immunology, SUNY Upstate Medical University, 766 Irving Ave Weiskotten Hall Suite 2281, Syracuse, NY 13210 USA; 2 Department of Pathology, SUNY Upstate Medical University, Syracuse, NY 13210 USA; 3 Department of Pediatrics, Rutgers Robert Wood Johnson Medical School Rutgers Child Health Institute of New Jersey, New Brunswick, NJ 08901 USA; 4 National Institute On Aging-National Institutes of Health, BRC Building, 251 Bayview Boulevard, Suite 100, Baltimore, MD 21224 USA; 5 Center On Aging and Immune Remodeling and Immunology Program, Department of Medicine, Johns Hopkins School of Medicine, Baltimore, MD 21224 USA

**Keywords:** NKG2D, GVHD, GVL, TCF-1, Eomes and T-bet

## Abstract

**Abstract:**

Cancer immunotherapy relies on improving T cell effector functions against malignancies, but despite the identification of several key transcription factors (TFs), the biological functions of these TFs are not entirely understood. We developed and utilized a novel, clinically relevant murine model to dissect the functional properties of crucial T cell transcription factors during anti-tumor responses. Our data showed that the loss of TCF-1 in CD8 T cells also leads to loss of key stimulatory molecules such as CD28. Our data showed that TCF-1 suppresses surface NKG2D expression on naïve and activated CD8 T cells via key transcriptional factors Eomes and T-bet. Using both in vitro and in vivo models, we uncovered how TCF-1 regulates critical molecules responsible for peripheral CD8 T cell effector functions. Finally, our unique genetic and molecular approaches suggested that TCF-1 also differentially regulates essential kinases. These kinases, including LCK, LAT, ITK, PLC-*γ*1, P65, ERKI/II, and JAK/STATs, are required for peripheral CD8 T cell persistent function during alloimmunity. Overall, our molecular and bioinformatics data demonstrate the mechanism by which TCF-1 modulated several critical aspects of T cell function during CD8 T cell response to cancer.

**Graphical abstract:**

Summary Figure: TCF-1 is required for persistent function of CD8 T cells but dispensable for anti-tumor response. Here, we have utilized a novel mouse model that lacks TCF-1 specifically on CD8 T cells for an allogeneic transplant model. We uncovered a molecular mechanism of how TCF-1 regulates key signaling pathways at both transcriptomic and protein levels. These key molecules included LCK, LAT, ITK, PLC-*γ*1, p65, ERK I/II, and JAK/STAT signaling. Next, we showed that the lack of TCF-1 impacted phenotype, proinflammatory cytokine production, chemokine expression, and T cell activation. We provided clinical evidence for how these changes impact GVHD target organs (skin, small intestine, and liver). Finally, we provided evidence that TCF-1 regulates NKG2D expression on mouse naïve and activated CD8 T cells. We have shown that CD8 T cells from TCF-1 cKO mice mediate cytolytic functions via NKG2D.
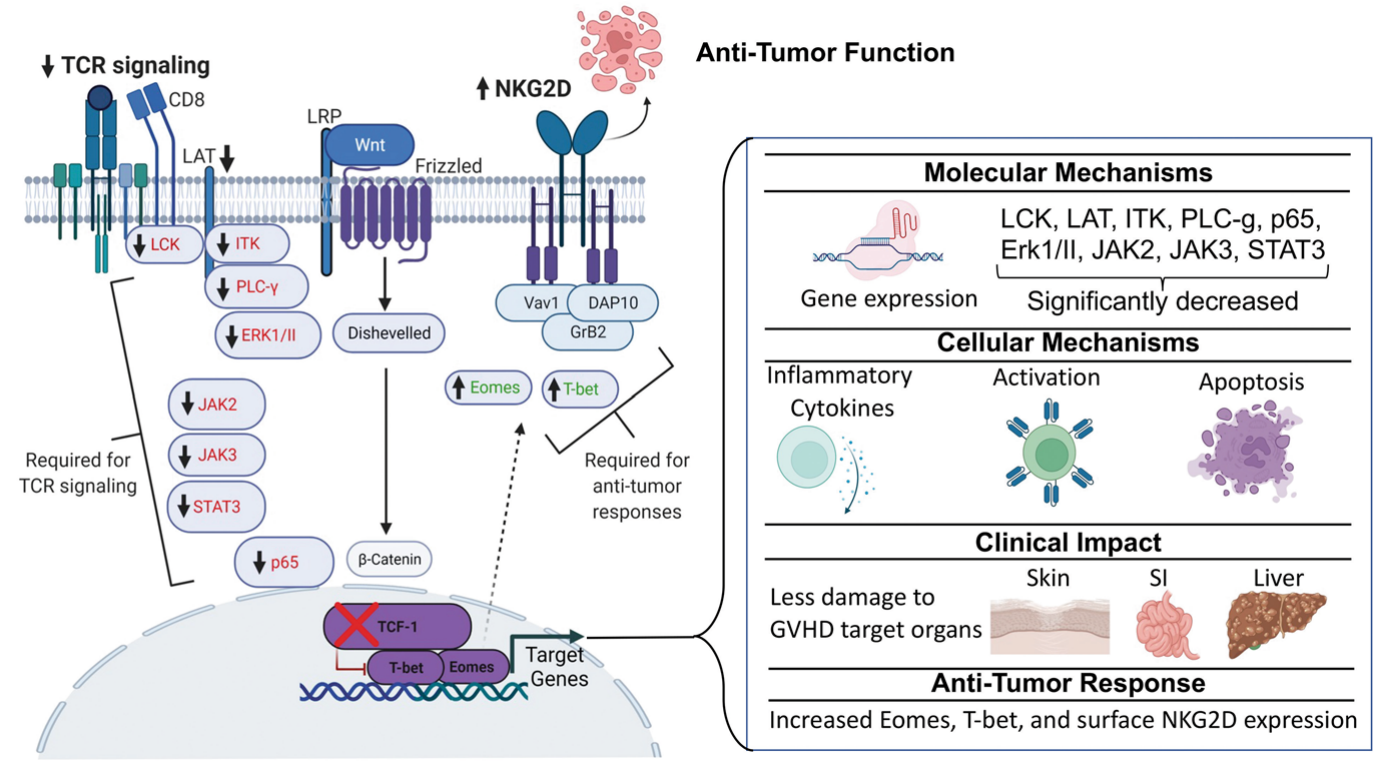

**Supplementary Information:**

The online version contains supplementary material available at 10.1007/s00262-022-03323-0.

## Introduction

T Cell Factor-1 (*TCF-1*), a major T cell developmental transcription factor, is involved in the Wnt signaling pathway, and is critical for T cell development as well as activation [1, 2]. Dysfunction of the Wnt/β-catenin/TCF-1 signaling pathway leads to immune deficiency or autoimmunity [3]. It is well known that TCF-1 is involved in the regulation of cell proliferation and survival during later T cell development [4]. In the absence of TCF-1, T cell development is completely blocked after early thymocyte progenitor (ETP) cells, which suggests that TCF-1 is also important in T cell lineage specification [5, 6]. TCF-1—Eomes^hi^ CD8 T cells are not proliferative and express higher levels of immune checkpoint receptors but maintain effector function [7–9]. Studies have also reported that TCF-1 + CD8 T cells with stem-like abilities express low levels of PD-1 and Tim checkpoint receptors, further providing evidence that TCF-1 is required for CD8 T cell formation and persistent function [10–12]. Studies have demonstrated that dysfunctional virus-specific CD8 T cells transplanted into naïve mice give rise to TCF-1-negative CD8 T cells [12]. However, tumor-specific CD8 T cells transferred to naïve mice might give rise to TCF-1 + CD8 T cells, demonstrating the differential role of TCF-1 in viral infection and tumor [13]. These differences could be due to the differences between microenvironments in viral infection and in tumors [14]. Whether TCF-1+ cells will give rise to TCF-1+ or TCF-1—cells will be dependent on internal and external signaling. Some studies have shown that both the long (p45) and the short (p33) TCF-1 isoform are expressed by CD8 T cells that will give rise to stem-like CD8 T cells during viral infection, and it has been shown that the long isoform of TCF-1 is capable of promoting stem-like CD8 T cell formation during viral infection by regulating genes like CD127, CXCR5, and cMyb [15]. Currently, the role of long and short TCF-1 isoforms is largely unknown.

Studies of T cells as immunotherapy in both human and mice showed that for a superior anti-tumor response, less differentiated cells are more favorable than a more differentiated subset of CD8 T cells [16–18]. Ideal CD8 T cells for immunotherapy have been shown to exhibit stem-like abilities (such as those obtained by inducing ex vivo cell growth with IL-17, IL-15, IL-21) and higher expression of TCF-1, Eomes, and Bcl6 [19, 20]. Thus, the suitability of TCF-1 as a target for immunotherapy to clear viral infection and cancer might also indicate considerable consequences for autoimmune diseases.

To study the role of TCF-1 in a clinically relevant model, we utilized allogeneic hematopoietic stem cell transplantation (allo-HSCT). In allo-HSCT, mature peripheral donor T cells found in the graft become alloactivated upon recognition of host HLA as non-self. These T cell activities help to clear residual malignant cells, which is called the graft-versus-leukemia effect (GVL) [21–23]. On the other hand, alloactivated T cells also target healthy recipient tissues, an effect known as graft-versus-host disease (GVHD) [24]. We used a unique mouse strain which has deletion of TCF-1 in mature T cells, rather than a global deletion [25]. This TCF-1 flox/flox x CD4cre mouse experiences deletion of TCF-1 in all T cells at the double-positive phase of development, when all T cells express CD4 [26]. This allows us to overcome the severe T cell developmental defect that occurs in global TCF-1 deletion, as TCF-1 is critical for the double-negative stage of development [27].

Using a mouse model of GVHD and GVL following allo-HSCT, we were able to study all of the major T cell functions, as well as phenotype, clinical outcomes, and gene expression, in a single model. In this model, we transplanted CD8 T cells from either WT or TCF-1 cKO mice into irradiated BALB/c mice (H2K^b^H2K^d^) [28–30]. Using allogenic preclinical model, we have shown that CD8 T cells from TCF-1 cKO effectively clear tumor cells without inducing GVHD by producing significantly less inflammatory cytokines as proinflammatory cytokines are considered the hallmark of alloimmunity [31]. Our data also uncovered that CD8 T cells from TCF-1 cKO mice cause significantly less tissue damage in GVHD target organs [35, 36]. Molecular analysis showed that CD8 T cells from TCF-1 cKO mice showed a significant reduction in key molecules required for CD8 T cell persistent function [5, 22, 32]. CD8 T cells from TCF-1 cKO mice exhibited innate memory-like phenotype by upregulating CD122, CD44, and effector and central memory phenotypes in mature CD8 T cells critical for GVHD development [33, 34]. We also uncovered that CD8 T cells from TCF-1 cKO mice significantly upregulated Eomes and T-bet, two downstream transcription factors which are known to be involved in GVL [28, 35–37]. The loss of TCF-1 also led to upregulation of NK cell type 2 receptor (NKG2D). NKG2D, encoded by *Klrk1*, is an activating cell surface receptor that is predominantly expressed on Natural killer cells [38, 39]. NKG2D is abundantly present on all NK cells, NKT cells, and subsets of *γδ* T cells [40]. While naïve human CD8^+^ T cells express NKG2D, in mice CD8 T cells only upregulate NKG2D upon activation [41, 42]. Human and murine CD8 T cells mediate cytotoxicity by releasing cytotoxic granules containing Perforin and Granzyme B, resulting in the killing of transformed or malignant cells [43, 44]. Our data also showed that loss of TCF-1 led to the upregulation of Granzyme B on NKG2D + CD8 T cells which reveals how TCF-1 deficient T cells can kill the tumor cells. Our molecular and animal studies were also confirmed by transcriptomic analysis.

Altogether, our data demonstrate that loss of TCF-1 in mature murine CD8 T cells enhanced Eomes and T-bet expression and reduced TCR signaling, resulting in less severe GVHD. Our data also demonstrated that TCF-1-deficient CD8 T cells utilized NKG2D receptors to kill tumor targets. These findings will have a considerable impact on developing strategies to uncouple GVHD from GVL, and for developing therapeutic interventions for T cell-driven autoimmune disorders.

## Materials and methods

### Mouse models

For transplant, the following female donor mice were used: B6-Ly5.1 (CD45.1+ , “WT” or B6.SJL-Ptprca Pepcb/BoyCrl, 494 from Charles River), C57Bl/6 J (CD45.2+ , “WT,” 000,664 from Jackson Laboratories), or TCF-1 flox x CD4cre (referred to here as TCF-1 cKO in both C57Bl/6 J background [45], obtained from Dr. Jyoti Misra Sen at NIH by permission of Dr. Howard Xue, and bred in-house) [46]. These donor mice were age-matched to each other and to recipients as closely as possible. BALB/c female mice (CR:028 from Charles River, age 6–8 weeks or older) were used as recipient mice for transplant experiments, and Thy1.1 mice (B6.PL-Thy1a/CyJ, 000,406 from Jackson Labs) were used as recipient mice for chimera experiments.

### Allotransplant and tumor models

BALB/c recipient mice were irradiated with two doses of 400 cGy of X-rays (total dose 800 cGy) and rested for at least 12 h between doses. Mice were also rested for 4 h prior to transplantation. T cells (total CD3+ or CD8+) were separated from WT, CD4cre+/+ , and TCF-1 cKO spleens using CD90.2 or CD8 microbeads and LS columns (Miltenyi, CD8: 130-117-044, CD90.2: 130-121-278, LS: 130-042-401). 1 × 10^6^ donor cells (unless otherwise mentioned) were injected IV into the tail vein in PBS, along with 10 × 10^6^ WT bone marrow cells. Bone marrow was T cell depleted with CD90.2 MACS beads (130-121-278 from Miltenyi) and LD columns (130-042-901 from Miltenyi). For short-term experiments, at day 7 post-transplant, recipient mice were euthanized and serum, spleen, small intestine, or liver were collected, depending on the experiment. For GVHD and GVL experiments, recipient mice were also given 2 × 10^6^ luciferase-expressing B-cell lymphoma (A-20) [47]. Recipient mice were weighed, given a clinical score, and imaged using the IVIS 50 imaging system three times per week until day 70 or longer. Clinical scores were composed of scores for skin integrity, fur texture, posture, activity, diarrhea, and weight loss. Imaging was done by injecting recipients I.P. with D-luciferin to detect tumor cell bioluminescence. To produce mixed bone marrow chimeras, Thy1.1 mice were lethally irradiated and reconstituted with a 1:4 (WT: TCF-1 cKO) mixture of bone marrow cells (total 50X10^6^ cells), then rested for 9 weeks. At 9 weeks, tail vein blood was collected and checked by flow cytometry for CD45.1 and CD45.2 to ensure reconstitution with both donor cell types. At 10 weeks, mice were used for phenotyping experiments [29].

### Flow cytometry, sorting, and phenotyping

Splenocytes (or cells from other organs) were obtained from WT, CD4 cre, or TCF-1 cKO mice or recipient allotransplanted mice. Red Blood cells were lysed with RBC Lysis Buffer (00-4333-57 from eBioscience) to remove red blood cells if needed. Cells were then stained with extracellular markers for 30 min on ice in MACS buffer (1X PBS with EDTA and 4 g/L BSA) [29]. If intracellular markers were used, the cells were then fixed and permeabilized using the Fix/Perm Concentrate and Fixation Diluent from FOXP3 Transcription Factor Staining Buffer Set (eBioscience cat. No. 00-5523-00). The cells were then run on a BD LSR Fortessa cytometer and data were analyzed using FlowJo software v9 (BD Biosciences).

All antibodies were used at a 1:100 dilution. For FACS sorting, the same methods were applied, and cells were run on a BD FACS Aria III with cold-sorting blocks. Cells were sorted into sorting media (10% FBS in RPMI) or Trizol, depending on the experiment. Depending on the experiment, antibodies used were: anti-CD4 (FITC, PE, BV785, BV21), anti-CD8 (FITC, PE, APC, PerCP, Pacific Blue, PE/Cy7), anti-CD3 (BV605 or APC/Cy7), anti-H2Kb-Pacific Blue, anti-H2Kd-PE/Cy7, anti-CD122 (FITC or APC), anti-CD44 (APC or Pacific Blue), anti-CD62L (APC/Cy7), anti CD28 PE Cat200902 anti-TNF-*α*-FITC, anti-IFN-*γ*-APC, anti-Eomes (AF488 or PE/Cy7), anti-T-bet-BV421, anti-CD45.2-PE/Dazzle594, anti-CD45.1-APC, anti-Ki67 (PE or BV421), anti-PD1-BV785, anti-CTLA4-PE, NKG2D-BV711, Granzyme B-PE/Cy7 [28–30, 35].

### Histology

Recipient mice were allotransplanted as described, and organs were removed for histology at day 7, and day 21 post-transplant. Spleen, liver, small intestine, and skin (from ear and back) were fixed, sectioned, and stained with H&E at Cornell University (https://www.vet.cornell.edu/animal-health-diagnostic-center/laboratories/anatomic-pathology/services). A pathologist (L.S) analyzed the sections for T cell-induced damage.

### Cytokine restimulation

Recipient BALB/c mice were allotransplanted with 1.5X10^6^ CD3 donor T cells and euthanized at day 7. Splenocytes were taken and cultured for 6 h with GolgiPlug (1:1000) and PBS (control) or anti-CD3 (1 ug/mL)/anti-CD28 (2 ug/mL) (TCR stimulation) at 37 C and 7% CO_2_. After 6 h of culture, the cells were stained for CD3, CD4, CD8, H2K^b^, TNF-*α*, and IFN-*γ* using the BD Cytokine Staining kit (BD Biosciences, 555,028), and run on a flow cytometer [28, 30, 35].

### LEGENDPlex serum ELISA assay

Serum from cardiac blood was collected from recipient mice in the cytokine restimulation experiment. Serum was analyzed using the Biolegend LEGENDPlex Assay Mouse Th Cytokine Panel kit (741,043). This kit quantifies serum concentrations of: IL-2 (T cell proliferation), IFN-*γ* and TNF-*α* (Th1 cells, inflammatory), IL-4, IL-5, and IL-13 (Th2 cells), IL-10 (Treg cells, suppressive), IL-17A/F (Th17 cells), IL-21 (Tfh cells), IL-22 (Th22 cells), IL-6 (acute/chronic inflammation/T cell survival factor), and IL-9 (Th2, Th17, iTreg, Th9—skin/allergic/intestinal inflammation) [28, 29].

### Western blot

Splenocytes from WT, CD4 cre+/+,  or TCF-1 cKO donor mice were collected. CD8 T cells were separated using CD8 MACS beads. CD8 T cells were either stimulated with 2.5 ug/ml anti-CD3 (Biolegend #100,202) and anti-CD28 antibodies (Biolegend #102,115) for 10 min or left unstimulated. These cells were counted and lysed with RIPA Buffer (89,900 from Thermo Fisher) plus protease inhibitors (11,697,498,001 from Millipore Sigma) and phosphatase inhibitors (P5726-1ML and P0044-1ML from Millipore Sigma). The lysates were run on a Western blot and probed for Perforin (Cell Signaling Technology #3693), Granzyme B (Cell Signaling Technology #4275), LCK (Thermo Fisher PA5-34,653), ZAP70 (Cell Signaling Technology #3165), LAT (Cell Signaling Technology # 45,533), ITK (Thermo Fisher PA5-49,363), PLC*γ*1 (Cell Signaling Technology #2822), ERK1-2 (Cell Signaling Technology #9107), JAK 2(Cell Signaling Technology # 3230), JAK3 (Cell Signaling Technology #8863), STAT3 (Cell Signaling Technology #9139), p65-Rela (Cell Signaling Technology #4764), AKT (Cell Signaling Technology #9272), and β-actin (Cell Signaling Technology #4970). All the Western blots repeated at least three times and one representative of each protein and quantification is shown.

### qPCR analysis

To perform qPCR, BALB/c mice were allotransplanted as described (1 × 10^6^ CD3 donor T cells). Pre-transplant donor cells and post-transplant (day 7) spleen and liver cells from recipients were FACS-sorted to obtain CD8 donor cells. The cells were sorted into Trizol, RNA was extracted using chloroform (https://www.nationwidechildrens.org/ Document/Get/93327), and eluted using the Qiagen RNEasy Mini kit (74,104 from Qiagen). Concentration was checked with a spectrophotometer, and then, RNA was converted to cDNA with an Invitrogen Super Script IV First Strand synthesis System kit (18,091,050 from Invitrogen). Final cDNA concentration was checked with a spectrophotometer, and then, cDNA was mixed with TaqMan Fast Advanced Master Mix (4,444,557 from Invitrogen) at a 10 ng/µL cDNA concentration. This master mix was added to premade 96 well TaqMan Array plates with chemokine/chemokine receptor primers (Thermo Fisher, Mouse Chemokines and Receptors Array plate, 4,391,524). qPCR was performed in a Quant Studio 3 thermocycler, and data were analyzed using the Design and Analysis software v2.4 (provided by Thermo Fisher). Five separate recipient mice were sorted, and cells were combined to make one sample for qPCR testing per condition/organ.

### NKG2D expression and NKG2D-mediated cytotoxicity in CD8 T cells

To determine the NKG2D expression in CD8 T cells, we obtained splenocytes from WT and TCF-1 cKO mice and stimulated T cells with 2.5 ug/ml anti-CD3 (Biolegend #100,202) and anti-CD28 antibodies (Biolegend #102,115) for 24, 48, or 72 h in culture, or left them unstimulated. GolgiPlug (1:1000) was added to stimulated samples for each time point, and samples were incubated at 37 C and 7% CO_2_. After 6 h of culture, the cells were stained with LIVE/DEAD Aqua and for CD3, CD8, NKG2D, and Granzyme B using the BD Cytokine Staining kit (BD Biosciences, 555,028), and run on a flow cytometer. To assess the NKG2D-mediated cytotoxicity, we used luciferase-expressing A20 cells as target cells as described earlier. Effector cells (MACS-sorted CD8 T cells from TCF-1 cKO or WT mice) were incubated in 2.5 μg/ml anti-CD3 and anti-CD28 coated plates for 48 h to induce optimal NKG2D expression. Then, effector cells were added at 40:1 effector-to-target ratios and incubated at 37 °C for 4 h with the A20 cells. Anti-NKG2D antibody (10 μg/mL, Bio X Cell #BE0334) or rat IgG1 isotype control antibody (10 μg/mL, Bio X Cell #BE0334) was added and incubated for 30 min before washing and plating. Triplicate wells were averaged and percent lysis was calculated from the data using the following equation: % specific lysis = 100 × (spontaneous death bioluminescence—test bioluminescence)/(spontaneous death bioluminescence—maximal killing bioluminescence)[28, 36, 48–50].

### Exhaustion/activation assay

To determine the in vitro exhaustion and activation of CD8 T cells, we obtained splenocytes from WT and TCF-1 cKO mice and either activated them with 2.5 ug/ml anti-CD3 (Biolegend #100,202) and anti-CD28 antibodies (Biolegend #102,115) for 24, 48, or 72 h in culture, or left them unstimulated, and stained for CD3, CD8, Ki-67, Tox, and PD-1 markers. To assess exhaustion and activation of in vivo donor CD8 T cells, recipient mice were allotransplanted as before (1X10^6^ CD3 donor T cells) and euthanized at day 7. Lymphocytes were obtained from spleen, and stained for CD3, CD4, CD8, H2K^b^, TOX, Ki-67, and PD-1 markers.

### DNA extraction and PCR

Donor mice were genotyped using PCR on DNA extracted from ear punches. At 4 weeks of age, mice were ear punched, and DNA was extracted using the Accustart II Mouse Genotyping kit (95,135–500 from Quanta Biosciences). Standard PCR reaction conditions and primer sequences from Jackson Laboratories were used for CD4cre. For TCF-1, primer sequences and reaction conditions were obtained from Dr. Jyoti Misra Sen of NIH.

Primers used for CD4 cre+/+ genotyping are: Common primer: 5′- GTTCTTTGTATATATTGAATGTTAGCC; WT reverse primer: 5′-TATGCTAGGACAAGAATTGACA; and Mutant reverse primer: 5′-CTTTGCAGAGGGCTAACAGC. PCR conditions: Step 1. 94 °C for 2:00 min; Step 2. 94 °C, 20 s; Step 3. 65 °C, 15 s; Step 4. 68 °C, 10 s; Step 5. Go to step2, 10X; Step 6. 94 °C, 15 s; Step.7 60 °C, 15 s; Step.8 72 °C, 10 s; Step.9 Go to Step 6, repeat 28X; Step.10, 72 °C, 2 min; Step.11 10 °C, infinite hold.

Primers used for TCF-1 genotyping: Forward primer: 5′- AGCTGAGCCCCTGTTGTAGA, Reverse primer #1: 5′- TTCTTTGACCCCTGACTTGG, Reverse primer #2: 5′- CAACGA GCTGGGTAGAGGAG. PCR conditions for TCF-1 are: Step.1 94 °C, 2 min; Step.2 55 °C 30 s; Step3. 72 °C 1 min; Step4. Go to Step.2 repeat 38X; Step.5 72 °C 10 min, 12 °C infinite hold.

### RNA sequencing

Recipient mice were allotransplanted as before (1 × 10^6^ CD3 donor T cells), except that donor CD8 T cells were also FACS-sorted prior to transplant. A sample of sorted donor cells was also saved for pre-transplanted RNA sequencing in Trizol. At day 7 post-transplant, donor CD8 T cells were FACS-sorted back from recipient spleen of TCF-1 cKO and WT-transplanted mice. The cells were all sorted into Trizol, then RNA was extracted and prepped by the Molecular Analysis Core (SUNY Upstate, https://www.upstate.edu/research/facilities/molecular-analysis.php). Paired end sequencing was done with an Illumina NovaSeq 6000 system at the University at Buffalo Genomics Core (http://ubnextgencore.buffalo.edu). For data analysis, we used the statistical computing environment R (v4.0.4), the Bioconductor suite of packages for R, and R studio (v1.4.1106). We calculated the transcript abundance by performing pseudoalignment using the Kallisto (Bray et al. [51]) (version 0.46.2). Calculated Transcript per million (TPM) values were normalized and fitted to a linear model by empirical Bayes method with the Voom (Law et al. [52]) and Limma (Ritchie et al. [53]) R packages to determine Differentially expressed genes—DEGs (FDR < 0.1, after controlling for multiple testing using the Benjamini–Hochberg method). DEG’s were used for hierarchical clustering and heatmap generation in R. Gene ontology enrichment analysis was conducted using either the Function Annotation Chart tools using only GO-BP and KEGG terms in the Database for Visualization and Integrative Discovery (Huang da et al. [90]) DAVID enrichment scores > 1.3 are equivalent to a *P* value < 0.05. For gene set enrichment analysis (GSEA), we used Hallmark and C2 gene set collections of Molecular Signatures Database (MsigDB) and cluster Profiler package in R. Data will be deposited on the Gene Expression Omnibus (GEO) database for public access https://www.ncbi.nlm.nih.gov/geo/ GSE203167.

The RNAseq experiment described here was performed as part of the experiment described in other recent publications from our laboratory [28–30, 35]. Therefore, the data generated for WT pre- and post-transplanted samples (CD4 and CD8) are the same as that shown in the papers mentioned, but here, these data are compared to data for *Cat-Tg* mice [30].

### Statistical analysis

Unless otherwise noted in the figure legends, all numerical data are presented as means and standard deviations with or without individual points. Analysis was done in GraphPad Prism v7 or v9. Most data were analyzed with Student’s t test, one-way ANOVA, or two-way ANOVA, with Tukey’s multiple comparisons test for ANOVA methods, depending on the number of groups. Kaplan–Meier survival analyses were done for survival experiments. All tests were two-sided, and *P* values less than 0.05 were considered significant. Transplant experiments used 3–5 mice per group, with at least two repeats. Ex vivo experiments were done two to three times unless otherwise noted with at least three replicates per condition each time. RNA seq was done once with three replicates per group and condition. qPCR was done once with one sample per condition, and five mice were combined to make the one sample. Western blots were done three times for unstimulated and 10 min-anti-CD3/CD28 stimulated samples, one experiment each is shown [28–30, 35, 50].

## Results

### Loss of TCF-1 in donor CD8 T cells reduced severity and persistence of GVHD symptoms, increased survival from lethal GVHD, and retained anti-tumor capabilities for the GVL effect

Most of the previous research on TCF-1 utilized a global TCF-1 knockout because the primary focus was on TCF-1’s role as a developmental factor [6, 32]. However, we wanted to study the role of TCF-1 in mature T cells. Global loss of TCF-1 results in minimal T cell production from the thymus, because TCF-1 is critical for DN stages of development [54]. Therefore, we obtained mice with a T cell-specific knockout for TCF-1 (TCF-1 flox/flox x CD4cre, called TCF-1 cKO here [25, 46, 55]. This allowed us to study mature T cells that developed normally in the thymus, and then lost expression of TCF-1 at the DP phase [55].

**Fig. 1:**
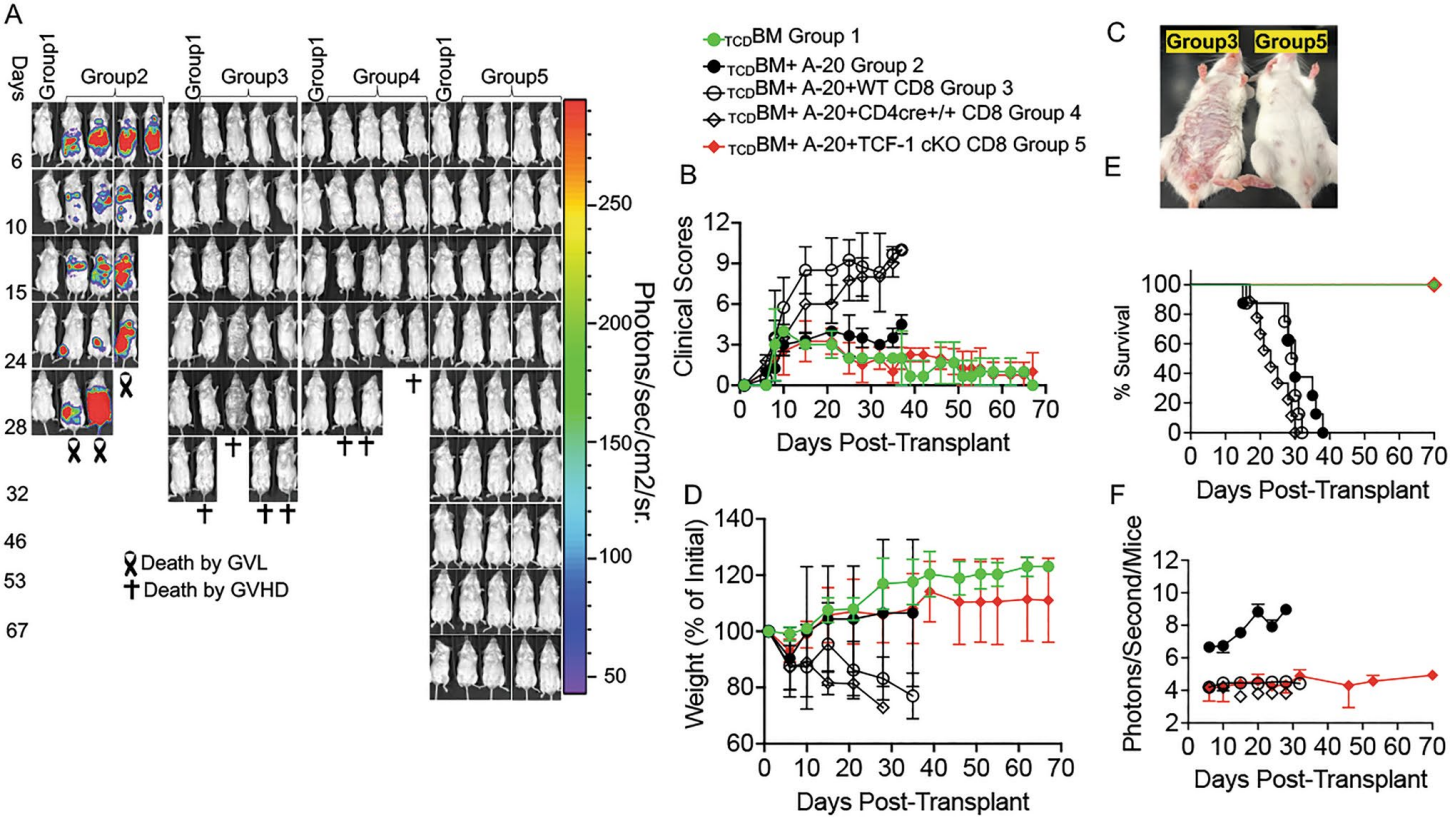
Loss of TCF-1 in donor CD8 T cells reduces severity and persistence of GVHD symptoms. BALB/c recipient mice (MHC haplotype d) were lethally irradiated and allotransplanted with 1 × 10^6^ CD8 T cells from WT, CD4 cre+/+,  or TCF-1 cKO donor mice (MHC haplotype b), as well as 10 × 10^6^ T cell-depleted bone marrow cells (BM) from WT mice (MHC haplotype b). Recipient mice were also given 2 × 10^5^ luciferase-expressing A20 tumor cells **A** The recipient mice were imaged 1 time a week using IVIS50 for 70 days. Gross pictures of representatives of recipients transplanted with CD8 T cells from WT, CD4 cre+/+,  and TCF-1 cKO mice at day 25 post-transplant are shown. **B** Recipient mice were also given a GVHD clinical score three times per week until day 70, based on combined scores of fur texture, activity level, weight loss, posture, skin integrity, and diarrhea. Mean and SD are plotted, analyzed by one-way ANOVA. **C** A representative image comparing groups 3 and 5 **D** Weight changes of the recipients mice also were tracked over the time course of disease. **E** Survival for each group of recipient mice up to 70 days post-transplant, analyzed by Kaplan–Meier survival analysis. **F** Quantification of bioluminescence of tumor growth. For all graphs, *means *P* value ≤ 0.05, **means *P* value ≤ 0.01, ***means *P* value ≤ 0.001, and ****means *P* value ≤ 0.0001 (*n* = 3 mice/group for BM alone: *n* = 4 experimental mice/group for all other groups). Survival is a combination of two experiments. Note: Control mouse is one of the mice from bone marrow only transplanted group used as a negative control for BLI

To determine whether TCF-1 plays a role in mature alloactivated T cell regulation, which is currently unknown, we used a mouse model of MHC-mismatched allo-HSCT, leading to GVHD and GVL. Briefly, BALB/c mice (MHC haplotype d) were lethally irradiated and transplanted with wild-type (WT) bone marrow and C57Bl/6-background (MHC haplotype b) donor CD8 T cells [28, 30, 35]. The donor CD8 T cells came from wild-type (WT), CD4 cre+/+ or TCF-1-deficient (TCF-1 cKO) mice. Recipients were given 1X10^6^ CD8 T cells and 10 × 10^6^ WT T cell-depleted bone marrow cells, as well as 2 × 10^5^ luciferase-expressing B-cell lymphoma (A-20) cells [47, 56] to assess GVL responses [28, 30, 35]. A20 cells are syngeneic to BALB/c mice and allogeneic to C57BL/6 (B6) mice [47, 56], meaning that the cells will not be naturally rejected by the BALB/c hosts, but will be attacked by the transplanted donor T cells. The MHC haplotype mismatch between host and donor cells drives alloactivation of donor T cells, which in turn causes GVHD and GVL effects [57]. To examine disease severity, progression, and recipient mouse survival, recipient mice were weighed and given a clinical score three times per week following transplant, until about day 70 (Fig. 1A–F). The mice were scored based on six factors: skin integrity, fur texture, posture, activity level, weight loss, and diarrhea [58]. Since the A20 cells express luciferase (called A20 luc) [28, 35], allowing us to track them by injecting D-luciferin into the recipient mice and imaging them with an in vivo bioluminescence scanner (IVIS 50), the mice were scanned one time per week with IVIS 50 until the end of the experiment (Fig. 1A and F).

We found that mice who received WT donor CD8 T cells had a rapid increase in GVHD severity, with a high score being reached by day 14, suggesting severe GVHD (Fig. 1B). This high score was maintained, suggesting persistent disease, and reached a high peak score at day 40 when the recipient mice died of disease burden (Fig. 1B–C). Recipient mice transplanted with CD8 T cells from either WT or CD4 cre+/+ mice lost weight initially and were unable to regain much weight (Fig. 1D). Our data showed that recipient mice transplanted with CD8 T cells from either WT or CD4 cre+/+ mice lost weight, had increased GVHD scores, and died of GVHD within 30–35 days post-transplantation (Fig. 1E). In contrast, mice given TCF-1 cKO CD8 T cells had much better survival, lower peak and average clinical scores, minimal disease burden, and a gain in weight following the initial weight loss period (Fig. 1B–D). In addition, the clinical scores for TCF-1 cKO CD8 T cells transplanted mice quickly reduced to near-control levels following peak score at day 10 (Fig. 1B), suggesting that disease does not persist in these mice. Therefore, loss of TCF-1 in donor T cells led to reduced GVHD severity and persistence, with improved survival (Fig. 1E).

Regarding anti-tumor effects, we observed that over time, the group receiving only bone marrow and tumor cells showed a large increase in tumor growth (Fig. 1A, F), because no T cells were present to control the tumor cells. In contrast, most mice given CD8 T cells from any donor type along with the BM and A20 luc cells were able to clear the tumor cells by the end of the experiment (Fig. 1A, F). The GVL effect was maintained even in TCF-1 cKO-transplanted mice (Fig. 1A, F). Altogether, these data show that TCF-1 is dispensable for GVL effects, but critical for GVHD. Therefore, loss of TCF-1 in donor T cells provides a clinically optimal phenotype, where GVHD severity is reduced but beneficial GVL effects are maintained. Data presented here are representative of several experiments, and only Fig. 1E shows a combination of 2 experiments.

### Loss of TCF-1 drives changes to mature CD8 T cell phenotype which are primarily cell-*extrinsic*

**Fig. 2:**
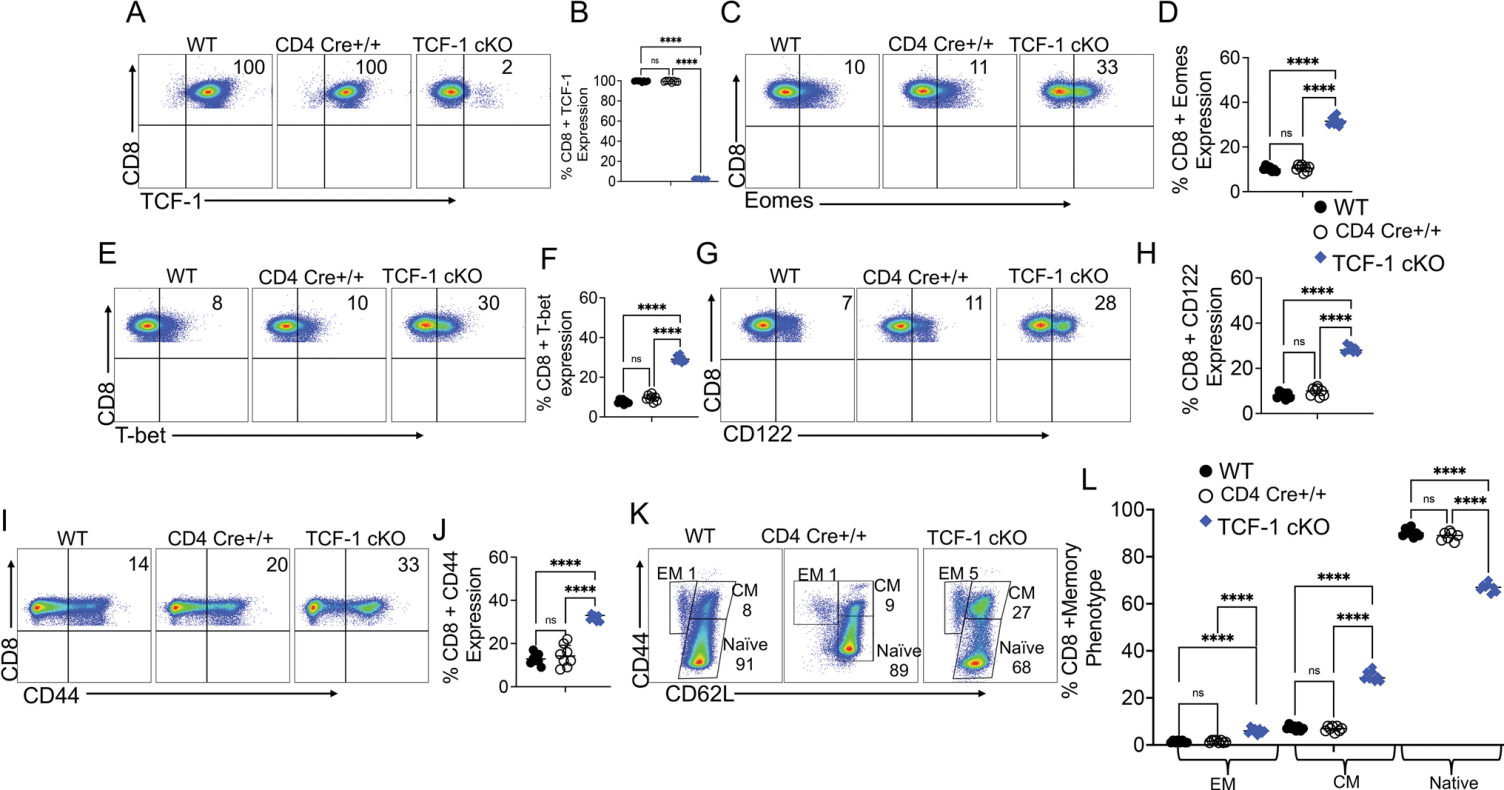
Loss of TCF-1 changes the mature CD8 T cell phenotype. Naïve WT, CD4 cre+/+,  and TCF-1 cKO donor mice were euthanized and splenocytes were stained for flow cytometry phenotyping. **A–B** Percent of CD8 T cells expressing TCF-1 and quantified statistical analysis **C–D** Percent of CD8 T cells expressing Eomes and quantified statistical analysis (**E–F**). Percent of CD8 T cells expressing T-bet and quantified statistical analysis. **G–H** Percent of CD8 T cells expressing CD122 and quantified statistical analysis. **I–J** Percent of CD8 T cells expressing CD44 and quantified statistical analysis. **K–L** Percent of CD8 T cells expressing central memory, effector memory, or naïve phenotypes and quantified statistical analysis. All data are plotted as individual points with mean and SD, all were analyzed with one-way ANOVA or Student’s t test (depending on groups). For all graphs, *means *P* value ≤ 0.05, **means *P* value ≤ 0.01, ***means *P* value ≤ 0.001, and ****means *P* value ≤ 0.0001. *N* = 2–3 per group per experiment, with combined data from three experiments shown

It has been shown that loss of TCF-1 in late stages of T cell development led to impaired output of CD4 T cells, and redirection of CD4 T cells to a CD8 T cell fate [55]. To determine whether loss of TCF-1 affected mature donor T cell phenotype, we performed flow cytometry phenotyping on CD8 T cells (Fig. 2). First, we confirmed the loss of TCF-1 expression in TCF-1 cKO mice by flow cytometry by examining TCF-1 expression on CD8 T cells from WT mice, CD4 cre+/+ mice, and TCF-cKO mice. In all of these experiments, cells were gated on lymphocytes, single positive CD3, and CD8+ T cells (Fig. 2A–B). Next, we examined whether the loss of TCF-1 also altered Eomesodermin (Eomes) and T-box transcription factor 21 (T-bet) expression, both of which are downstream of TCF-1 [15]. We examined Eomes, T-bet expression on freshly isolated CD8 T cells from WT, CD4 cre+/+ and TCF-1 cKO mice. Previously, experiments conducted using global TCF-1 cKO mice showed that Eomes is activated by TCF-1 (meaning loss of TCF-1 reduces Eomes expression) [58]. However, our model is unique compared to the global knockout mice, in that our mice are lacking TCF-1 expression specifically on mature CD8 T cells. Therefore, our TCF-1 cKO mice showed that CD8 T cells had increased expression of Eomes compared to WT CD8 T cells. In order to examine whether the increased Eomes expression could be the result of CD4cre, we examined CD8 T cells from CD4 cre+/+ mice. Our data demonstrated that the upregulation of Eomes and T-bet are the result of loss of TCF-1 and not affected by CD4cre (Fig. 2C–D). Reports using mice globally TCF-1 KO mice have claimed that T-bet may be activated or not affected by TCF-1 [2], but we found that loss of TCF-1 led to increased T-bet expression in mature CD8 T cells (Fig. E–F). This suggests that TCF-1 normally suppresses the expression of Eomes and T-bet in mature CD8 T cells.

Some reports have suggested that CD44^hi^ T cells do not cause GVHD or cause less severe GVHD [33]. Therefore, we wanted to examine CD8 T cells from TCF-1 cKO mice for activation markers like CD44 and CD122. Our data showed that CD8 T cells from TCF-1 cKO mice exhibit increased expression of CD122 and CD44 (Fig. 2G–J). Next, using CD62L and CD44 markers, we identified three major memory subsets: central memory (CD44^hi^ CD62L^hi^), effector memory (CD44^hi^ CD62L^low^), and naïve (CD44^low^ CD62L ^hi^) cells (Fig. 2K–L). TCF-1 cKO mice showed increased central memory CD8 T cell subsets and decreased naïve CD8 T cells (Fig. 2K–L). Thus, loss of TCF-1 results in a more memory-skewed phenotype for CD8 T cells. Some reports have suggested that memory T cells delay induction of GVHD [33, 35], so this phenotypic change may be beneficial [59].

Changes to cell phenotype in a knockout mouse may be cell-intrinsic (due directly to gene deficiency within the cell) or cell-extrinsic (due to changes in the microenvironment from gene deficiency) [60]. To determine whether the phenotypic effects we observed were cell-intrinsic or cell-extrinsic, we developed a chimeric mouse model. Briefly, we mixed bone marrow from WT and TCF-1 cKO mice at a 1:4 (WT:TCF) ratio for a total of 50X10^6^ BM cells, then used this mixture to reconstitute lethally irradiated Thy1.1 mice. We used a 1:4 ratio based on our previous published work [28, 35], to ensure survival of the KO cells (based on our initial observations that TCF-1 cKO T cells did not proliferate well in culture). At 9 weeks post-transplant, blood was taken to ensure reconstitution and survival of both donor types in each mouse. At 10 weeks, splenocytes were phenotyped by flow cytometry, with donor cells being identified by H2K^b^, CD45.1 (WT), and CD45.2 (TCF-1 cKO) markers [28, 35].

First, we looked at the TCF-1 expression in CD45.1+ (WT), and CD45.2+ (TCF-1 cKO) cells and confirmed that cells from TCF-1 cKO mice did not express TCF-1 in chimeric mice (Supp. Fig. 1A). We did see a statistically significant increase in T-bet expression in CD8 T cells from TCF-1 cKO donor cells compared to WT donor cells in chimeric mice, when we performed a t test (data not shown). However, when we compared the T-bet expression in chimeric versus naïve CD8 T cells, we observed that T-bet expression in CD8 T cells from *TCF-1* cKO donor mice was reduced to near-WT levels from elevated levels (Supp. Figure 1B). This suggests that the increased expression of T-bet seen in TCF-1 cKO CD8 T cells from naïve mice is a cell-extrinsic effect. Interestingly, in the chimeric mice we observed that Eomes and CD122 expression levels in WT CD8 T cells were significantly increased to near-TCF-1 cKO levels, suggesting that the increase in Eomes and CD122 expression in CD8 T cells from TCF-1 cKO mice is primarily cell-intrinsic (Supp. Fig. 1C-D).

Next, we examined the expression of CD44 and central memory phenotype in chimeric mice. We observed that while the frequencies of these subsets were lower in TCF-1 cKO-derived CD8 T cells compared to WT-derived CD8 T cells in the chimera (opposite of the trend observed in naïve mice), this was because the frequencies of CD44 and CM phenotype in WT cells were enhanced to the levels expressed by TCF-1 cKO cells from naïve mice (Supp. Figure 1E-F). These results suggest that the effects of TCF-1 deficiency on CD44 and CM phenotype expression in naïve mice could be primarily cell-intrinsic, with cell-extrinsic elements as well. Interestingly, effector memory phenotype in the chimeric mice we observed that levels in WT CD8 T cells were significantly increased to near-TCF-1 cKO levels, suggesting that the increase in effector memory phenotype in CD8 T cells from *TCF-1* cKO mice is primarily cell-intrinsic (Supp. Figure 1G). Finally, the naïve CD8 T cell population in the chimera coming from TCF-1 cKO bone marrow was significantly increased compared to CD8 T cells from WT bone marrow and compared to naïve TCF-1 cKO mice (Supp. Figure 1H). This suggests that the effect on naïve CD8 T cells in TCF-1 cKO mice could be either cell-intrinsic or cell-extrinsic. Altogether, these data suggest that the phenotypic changes seen in TCF-1 cKO may be primarily cell-intrinsic, with some additional cell-extrinsic effects being present.

### Loss of TCF-1 abolished CD28 expression and led to utilization of NKG2D to mediate *cytotoxicity*

Our data demonstrated that the loss of TCF-1 increases Eomes and T-bet expression in mature CD8 T cells (Fig. 2B–C). Considering that Eomes and T-bet have been reported to play a central role in anti-tumor responses, we hypothesized that by upregulating Eomes and T-bet, CD8 T cells lacking TCF-1 can maintain cytotoxicity, and that TCF-1 is not required for CD8 T cell-mediated cytolytic function [61]. We anticipated that CD8 T cells from TCF-1 cKO mice may have attenuated TCR signaling.

**Fig. 3:**
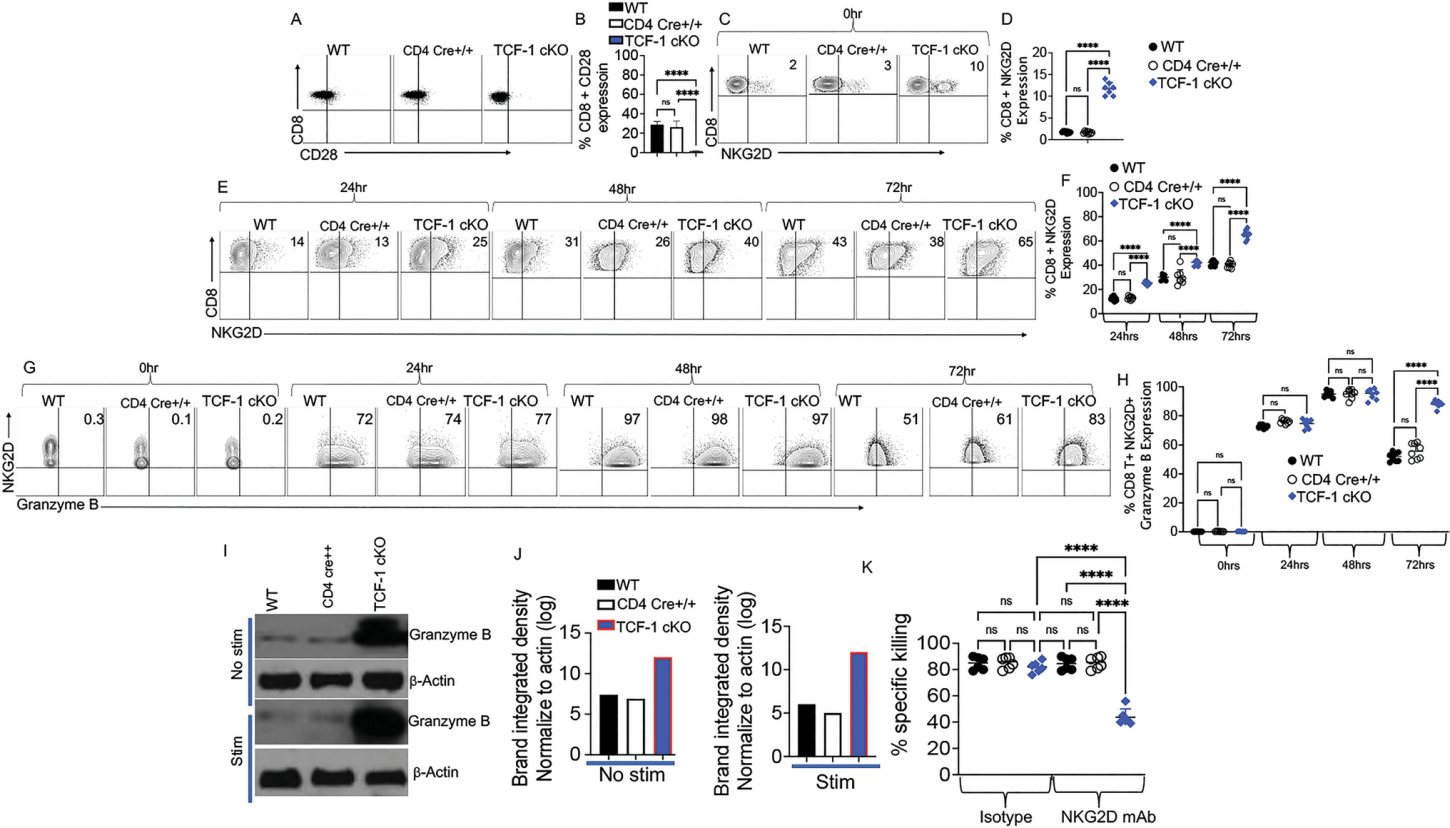
Loss of TCF-1 abolish CD28 expression and CD8 T cells from TCF-1 cKO mice utilize NKG2D to mediate cytotoxicity. Total splenocytes were isolated from WT, CD4 cre+/+,  or TCF-1 cKO mice and either left unstimulated or stimulated with anti-CD3/CD28 for 24, 48, or 72 h in culture. GolgiPlug (1:1000) was added to stimulated samples in each time point except 0-h samples, and samples were incubated at 37 C with 7% CO2. After 6 h of culture, the cells were stained with LIVE/DEAD Aqua, and for CD3, CD8, NKG2D, and Granzyme B expression, as determined by flow cytometry. **A–B** CD8 T cells from WT, CD4 cre+/+,  or TCF-1 cKO mice were examined for CD28 expression. **C–D** Percent of NKG2D expression in freshly isolated CD8 T cells from WT, CD4 cre+/+,  or TCF-1 cKO mice and quantified statistical analysis. **E–F** Percent of NKG2D expression in 24, 48, or 72 h-stimulated CD8 T cells from WT, CD4 cre+/+,  and TCF-1 cKO mice, and quantified statistical analysis. **G–H** Percent of Granzyme B expression in unstimulated or 24, 48, or 72 h-stimulated CD3+ CD8+ NKG2D+ T cells from WT, CD4 cre+/+, and TCF-1 cKO mice, and quantified statistical analysis. **I–J** Granzyme B expression in unstimulated CD8 T cells from WT, CD4 cre+/+,  and TCF-1 cKO mice by Western blot, and bands’ integrated density normalized to β-actin (quantified). Granzyme B expression in 10 min-anti-CD3/CD28-stimulated CD8 T cells from WT and TCF-1 cKO mice by Western blot, and bands’ integrated density normalized to β-actin (quantified) **K** To assess the NKG2D-mediated cytotoxicity, we used luciferase-expressing A20 cells as target cells. Effector cells (MACS-sorted CD8 T cells from WT, CD4 cre+/+,  or TCF-1 cKO mice) were incubated in 2.5 μg/ml anti-CD3 and anti-CD28 coated plates for 48 h to induce optimal NKG2D expression. Then effector cells were added at 40:1 effector-to-target ratios and incubated at 37 °C for 4 h with A20 cells. Anti-NKG2D antibody (10 μg/mL) or rat IgG1 isotype control antibody (10 μg/mL) was added and incubated for 30 min before washing and plating. Triplicate wells were averaged, and percent lysis was calculated from the data using the following equation: % specific lysis = 100 × (spontaneous death bioluminescence—test bioluminescence)/(spontaneous death bioluminescence—maximal killing bioluminescence) [74, 82, 83]. *N* = 4 per group with one representative of two experiments shown. Western blots were repeated twice and representative examples are shown. All data are shown as individual points with mean and SD, and were analyzed with Student’s t test or one-way ANOVA (depending on groups). *means *P* value ≤ 0.05, **means *P* value ≤ 0.01, and ***means *P* value ≤ 0.001

Ligation of the CD28 receptor on T cells provides a critical second signal alongside T cell receptor (TCR) ligation for naïve T cell activation. Our data supported by several publications showed that TCF-1 is critical for TCR stemness [1, 62]. Published data have also demonstrated that the lack of CD28 significantly weakens TCR stemness [63]. Thus, we examined whether the CD8 T cells from TCF-1 cKO mice also have reduced CD28 expression. We isolated CD8 T cells from WT, CD4 cre+/+,  and TCF-1 cKO mice. These freshly isolated ex vivo cells were examined for CD28 expression by flow cytometry. Our data uncovered that CD8 T cells from TCF-1 cKO mice abolished CD28 expression compared to the WT and CD4 cre+/+ mice (Fig. 3A–B). CD8 T cells can utilize either CD28 or NKG2D as co-stimulatory receptors [42, 64–66]. Studies have also shown that CD28^−^ CD8 T cells have higher NKG2D receptor expression [67]. Thus, we examined whether the lack of CD28 expression on CD8 T cells might lead to utilization of NKG2D as a co-stimulatory receptor. Our other rationale for examining NKG2D expression was that it is also known that Eomes and T-bet overexpression increases NKG2D expression in NK cells [68]. Considering that loss of TCF-1 in mature T cells led to upregulation of Eomes and T-bet expression, we hypothesized that loss of TCF-1 may also lead to upregulation of NKG2D expression in CD8 T cells and enhance the anti-tumor response.

Natural killer group 2 member D (NKG2D) is constitutively expressed on mouse NK cells, NKT cells, and some other cells [69], but does not get expressed on naïve mouse CD8 T cells [70]. Human CD8 T cells always express NKG2D on their surface, but mouse CD8 T cells only express it upon activation [39]. NKG2D is activated by NKG2D ligands [71, 72], and NKG2D ligands are relatively restricted to malignant or transformed cells [71, 72]. In order to determine whether loss of TCF-1 affects NKG2D expression and anti-tumor responses, we analyzed NKG2D expression in CD8 T cells. We measured NKG2D expression by flow cytometry before and at different time points after CD3/CD28 activation [50]. We found that CD8 T cells from TCF-1 cKO mice had significantly increased expression of NKG2D on the cells surface compared to CD8 T cells from either WT or CD4 cre+/+ mice, before stimulation (Fig. 3C–D). Next, we wanted to examine whether NKG2D expression was further upregulated on CD8 T cells from TCF-1 cKO mice compared to CD8 T cells from either WT or CD4 cre+/+ mice after stimulation. CD8 T cells were cultured with 2.5ug/ml anti-CD3 and 2.5ug/ml anti-CD28 antibodies for 24, 48, or 72 h. These cultured cells were examined for NKG2D expression by flow cytometry. Cells were gated on live cells, lymphocytes, and CD3/CD8 T cells. We observed an increase in NKG2D expression on CD8 T cells from WT CD4 cre+/+ and TCF-1 cKO mice in a time-dependent manner, and at all time points, expression of NKG2D was higher for cells from TCF-1 cKO mice (Fig. 3E–F). There was no difference in the viability of the cells or CD8 T cell numbers before or after the culture (Supp. Fig. 2A–B). We and others have shown that both human and murine CD8 T cells mediate cytotoxicity by releasing cytotoxic granules containing Granzyme B, resulting in the killing of transformed or malignant cells [43, 44]. Therefore, we also wanted to compare the Granzyme B expression in CD8+ , NKG2D+ T cells from WT, CD4 cre+/+,  and TCF-1 cKO mice [73]. First, cells in these experiments were gated on lymphocytes, single positive CD3+ , CD8+ . Next, we gated cells for NKG2D and Granzyme B positive. We did not observe any Granzyme B expression in CD8 T cells before stimulation (Fig. 3G–H). Only 24 h after stimulation, we observed Granzyme B expression in T cells from both strains, peaking at 48 h post-stimulation with no difference between strains of mice (Fig. 3G–H). After 72 h post-stimulation, CD8 T cells from WT, CD4 cre+/+ mice had significantly reduced Granzyme B expression compared to TCF-1 cKO CD8 T cells (Fig. G–H). We also confirmed total Granzyme B expression in CD8 T cells from WT, CD4 cre + / + , and TCF-1 cKO mice, in the presence and absence of CD3/CD28 stimulation, using Western blotting. Total Granzyme B expression was upregulated in CD8 T cells from TCF-1 cKO mice compared to WT, CD4 cre+/+ mice (Fig. 3I–J). These data demonstrated that CD8 T cells from TCF-1 cKO mice may maintain anti-tumor responses by killing the target cells with an NKG2D-mediated mechanism, and by persistent upregulation of Granzyme B expression [74, 75].

Next, we wanted to examine the functional consequences of upregulation of NKG2D expression on CD8 T cells from TCF-1 cKO mice. We utilized an in *vitro* cytotoxicity assay, where we used anti-NKG2D neutralizing antibody. We isolated CD8 T cells from WT, CD4 cre+/+,  and TCF-1 cKO mice and cultured them for 48 h with anti-CD3/anti-CD28 antibodies in order to induce optimal NKG2D expression in CD8 T cells. CD8 T cells from TCF-1 cKO and WT mice were then cultured with tumor target A20 cells [47] in a 40:1 ratio of tumor cells to CD8 T cells, along with anti-NKG2D antibody or isotype control antibody for 4 h. We used the A20 cell line as a tumor target because we previously showed that A20 cell line express NKG2D ligands including Rae1, H60, and MULT1 [76]. NKG2D-L can be also upregulated by viral infection, transformation, and stress [39, 77]. Triplicate wells were averaged, and percent lysis was calculated from the data using the following equation: % specific lysis = 100 × (spontaneous death bioluminescence—test bioluminescence)/(spontaneous death bioluminescence—maximal killing bioluminescence) [48].

Our data showed that the addition of anti-NKG2D antibody significantly reduced the cytotoxicity of CD8 T cells from TCF-1 cKO mice, whereas addition of isotype control had no effect on cytotoxicity of the CD8 T cells from TCF-1 cKO mice (Fig. 3K). In contrast, the addition of anti-NKG2D antibody [50] did not change cytotoxicity of the CD8 T cells from WT mice (Fig. 3K). It has been also shown that CD8 T cells from TCF-1 cKO mice lose CD28 expression, and cells with weaker TCR signaling have been shown to utilize NKG2D for cytolytic function [49, 50, 76, 78–80]. These data further support the idea that TCF-1 cKO CD8 T cells maintain their anti-tumor activity through an NKG2D-mediated mechanism. Taking into account that normal tissue does not express NKG2D ligands on the surface and that primarily malignant and transformed cells upregulate these ligands, this could explain why CD8 T cells from TCF-1 cKO mice cause less GVHD but maintain their anti-tumor activity [76]. These data further provided evidence that TCF-1 regulates stemness for TCR, and that lack of TCF-1 in CD8 T cells might activate the cells to clear tumor, but due to the lack of persistence, CD8 T cells from TCF-1 cKO mice do not induce GVHD.

### Loss of TCF-1 alters cytokine production, chemokine expression, and expression of exhaustion markers by mature CD8 T cells

We confirmed that CD8 T cells from TCF-1 cKO mice mediate cytolytic function primarily through NKG2D. Next, we wanted to examine the mechanism behind why CD8 T cells from TCF-1 cKO mice induce less GVHD. One of the hallmarks of GVHD is the release of proinflammatory cytokines by alloactivated donor T cells, eventually leading to cytokine storm [81, 82]. We examined whether loss of TCF-1 in donor CD8 T cells led to changes in cytokine production, thereby affecting GVHD damage. We allotransplanted lethally irradiated BALB/c mice as described above. Recipient mice were transplanted with 1.5 × 10^6^ WT or TCF-1 cKO CD8 donor T cells. Recipients were sacrificed at day 7 post-transplant. Splenocytes were isolated and restimulated by 6 h of culture with PBS (control) or anti-CD3/anti-CD28 (stimulation), along with Golgiplug. Afterward, the cultured cells were stained with antibodies against H2K^b^, CD3, CD4, CD8, TNF-*α*, and IFN-*γ*. Our data showed that production of TNF-*α* by donor CD8 T cells trended toward decreasing when TCF-1 was lost (Supp. Figure 3A). In contrast, IFN-*γ* trended toward increasing upon loss of TCF-1 in CD8 T cells (Supp. Figure 3B).

**Fig.4:**
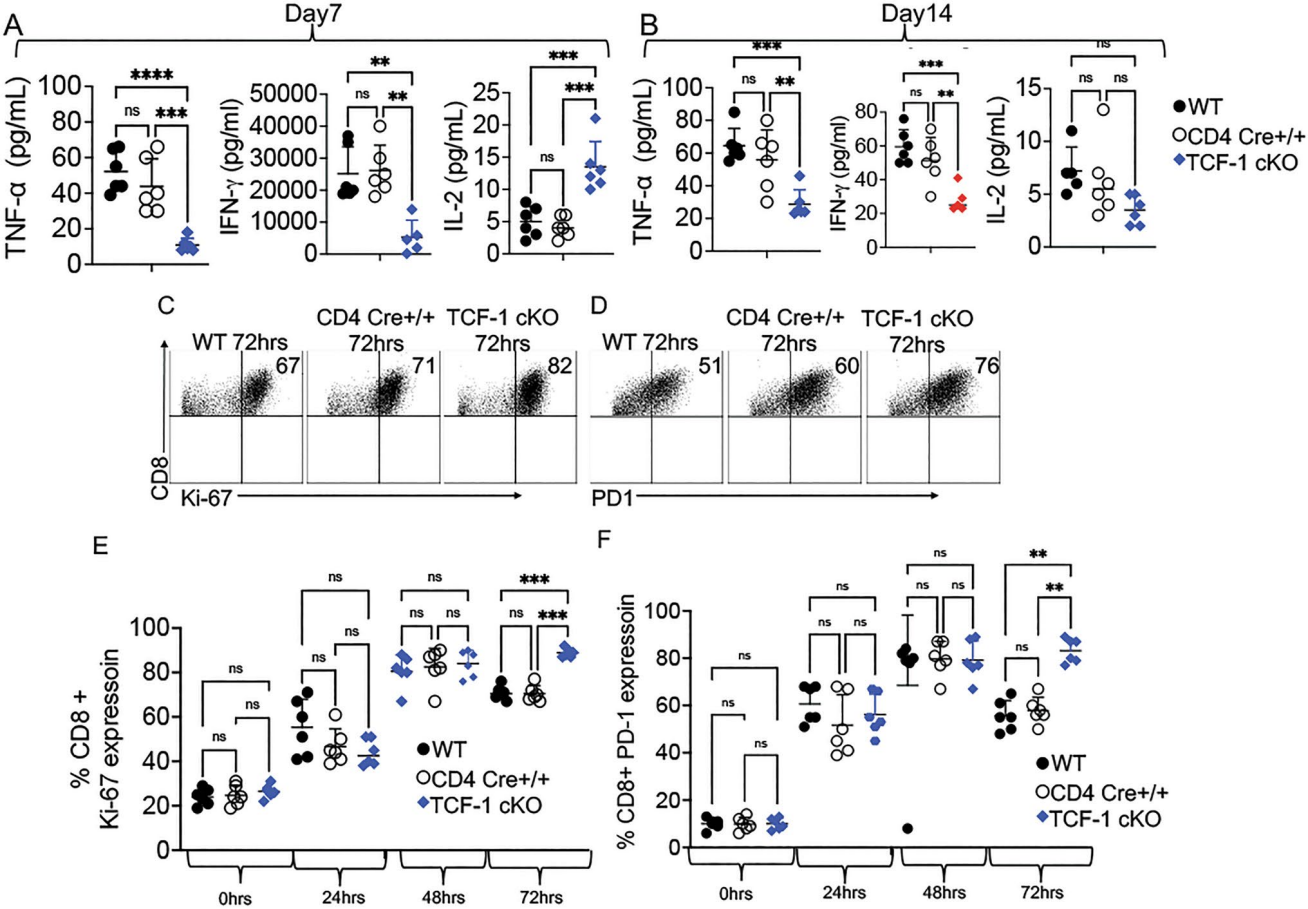
TCF-1 controls cytokine production and exhaustion of mature alloactivated CD8 T cells. **A–B** Recipient mice were allotransplanted with 1.5 × 10^6^ WT, CD4 cre+/+ or TCF-1 cKO donor CD3 T cells as before**.** At day 7 and day 14 post-transplant, serum was obtained from cardiac blood of allotransplanted recipient mice and tested for various cytokines using a LEGENDPlex ELISA assay. **A** Serum levels (pg/mL) of TNF-*α*, IFN-*γ*, and IL-2 for WT, CD4 cre+/+, and TCF-1 cKO-transplanted mice at day 7 post-transplant. **B** Serum levels (pg/mL) of TNF-*α*, IFN-*γ*, and IL-2 for WT, CD4cre+/+,  and TCF-1 cKO-transplanted mice at day 14 post-transplant. **C–F** Splenocytes from TCF-1 cKO, WT and CD4 cre+/+ mice were obtained and stimulated with anti-CD3/CD28 antibodies for 24 h, 48 h, or 72 h in culture and stained for Ki-67 and PD-1 or were stained immediately after isolation without stimulation. **C** Percent expression of Ki-67 in CD8 T cells after 72 h of anti-CD3/CD28 stimulation in culture determined by flow cytometry. **D** Quantification of the in vitro Ki-67 expression of CD8 T cells at different time points. **E** Percent expression of PD-1 in CD8 T cells after 72 h of anti-CD3/CD28 stimulation in culture determined by flow cytometry. **F** Quantification of the in vitro PD-1 expression of CD8 T cells are different time points**.** All data are shown as individual points with mean and SD, and were analyzed with Student’s t test or two-way ANOVA (depending on groups). *means *P* value ≤ 0.05, **means *P* value ≤ 0.01, and ***means *P* value ≤ 0.001

Levels of TNF-*α* and IFN-*γ* in serum of recipient mice given TCF-1 cKO CD8 T cells were lower than in mice given WT or CD4 cre+/+ CD8 T cells at day 7 (Fig. 4A). In contrast, the serum levels of IL-2 in mice given TCF-1 cKO CD8 T cells were higher than in mice given WT, CD4 cre+/+ CD8 T cells at day 7 (Fig. 4A). At day 14 post-transplant, the reduction in TNF-*α* and IFN-*γ* levels observed at day 7 for TCF-1 cKO-transplanted mice was preserved (Fig. 4B). We observed a trend toward decreased serum levels of IL-2 in mice given TCF-1 cKO CD8 T cells compared with mice given WT and CD4 cre+/+ mice. CD8 T cells at day 14 post-transplant, opposite of the effect observed on day 7 post-transplant (Fig. 4B). This suggests that TCF-1 cKO CD8 T cells may be capable of IFN-*γ* and TNF-*α* production at the same level as WT or CD4 cre+/+ CD8 T cells when restimulated, but in reality, produce less IFN-*γ* and TNF-*α* than WT or CD4 cre+/+ cells when allotransplanted.

In order for GVHD to persist, donor T cells must proliferate in secondary lymphoid organs and target organs [83, 84]. Naïve and effector T cells drive GVHD, but they are short-lived and must be replaced to maintain an alloresponse [85]. Also, given that memory cells are increased among CD8 T cells when TCF-1 is lost, we hypothesized that activation and/or exhaustion of these cells may also be affected. Ki-67 [86] is a marker of T cell activation and proliferation, and TOX [87] and PD-1 are markers of activation and exhaustion [88]. Therefore, we wanted to determine the Ki-67, TOX, and PD-1 expression levels on WT, CD4 cre+/+,  and TCF-1 cKO CD8 T cells in vitro. We cultured splenocytes from either WT, CD4 cre+/+ mice, or TCF-1 cKO mice in vitro with anti-CD3 and anti-CD28 antibodies for 24, 48, and 72 h. We did not observe any difference in Ki-67 expression in cells that were not stimulated, but the CD8 T cells from TCF-1 cKO mice that were stimulated for 72 h in vitro showed significant upregulation of Ki-67 expression (Fig. 4C–D), suggesting that CD8 T cells from TCF-1 cKO mice could potentially proliferate more than CD8 T cells from WT or CD4 cre+/+ mice when restimulated**.** We also observed the same trend of increased expression for PD-1 at 72 h post-stimulation (Fig. 4E–F). There were no differences in expression of TOX at any time points in vitro when CD8 T cells from TCF-1 cKO mice were compared to WT or CD4 cre+/+ (Supp. Fig. 3C).

Next, we checked the expression of these markers in vivo on donor cells that were allotransplanted in recipient Balb/c mice as described earlier. At day 7 post-transplant, splenocytes were isolated and Ki-67, TOX, and PD-1 expression were detected by flow cytometry. We did not observe differences in TOX expression on donor CD8 + T cells from TCF-1 cKO compared to WT or CD4 cre+/+ CD8 T cells (data not shown). We did not observe any statistically significant differences in Ki-67 or PD-1 expression at day 7 post-transplant (Supp. Fig. 3D-E). Taken together these data suggest that CD8 T cells from TCF-1 cKO mice could be more exhausted than WT or CD4 cre+/+ CD8 T cells both in vivo and in vitro.

One of the major functions of alloactivated T cells is migration from spleen to GVHD target organs, including liver and small intestine [84]. Expression of chemokines and chemokine receptors is a critical aspect of T cell migration to target organs. To determine whether expression of these molecules was affected by loss of TCF-1 in CD8 T cells, we FACS-sorted pre- and post-transplanted donor CD8 T cells from WT or TCF-1 cKO mice (spleen only for pre-transplant, spleen, or liver for post-transplant). We then extracted RNA from the cells, converted it to cDNA, and performed qPCR using a 96-well mouse chemokine/chemokine receptor plate (Thermo Fisher). As expected, expression of these markers was generally upregulated in alloactivated cells. Expression of these markers was generally higher in TCF-1 cKO CD8 T cells from pre-transplant spleen compared to WT pre-transplant spleen (Supp. Figure 4A), but these markers were generally downregulated in TCF-1 cKO CD8 T cells from post-transplant liver and spleen compared to WT cells (Supp. Figure 4B-C). Therefore, TCF-1 controls expression of CD8 T cell chemokine/chemokine receptors.

### Loss of TCF-1 in donor CD8 T cells led to decreased damage to the GVHD target organs

**Fig.5:**
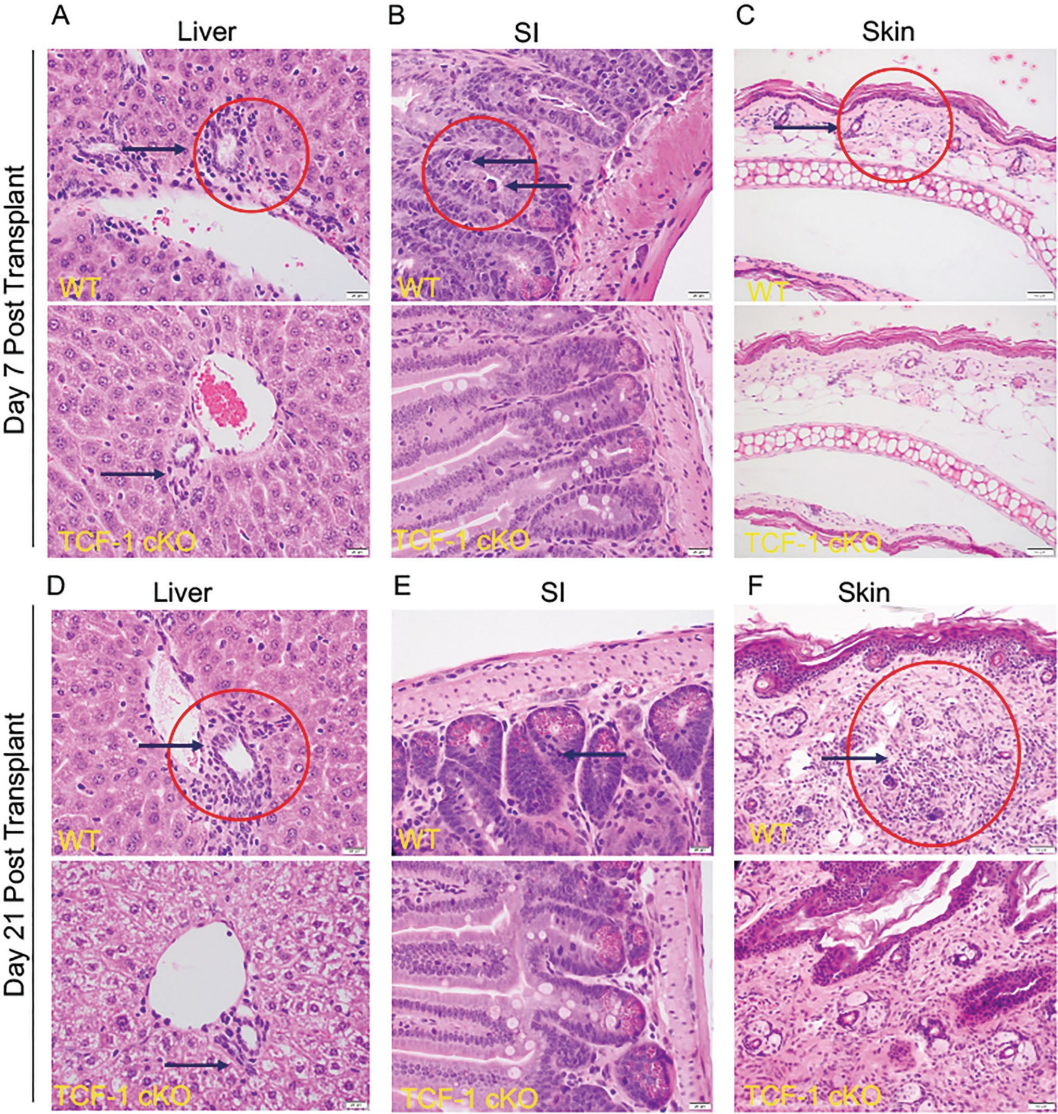
Loss of *TCF-*1 in donor CD8 T cells decrease the damage to the GVHD target organs. We collected organs from mice allotransplanted as described above. At day 7 and day 21 post-transplant, organs were taken from recipient mice for histology analyses. Skin, liver, spleen, and small intestine were sectioned, stained with H&E, and analyzed by pathologist. Representative sections for each organ per group and timepoint are shown. **A** H&E staining of the liver of the recipient mice at day 7 post-transplant (black arrows showing the interlobular bile ducts and red circle showing inflammatory infiltrates). **B** H&E staining of the small intestines of the recipient mice at day 7 post-transplant (black arrows showing the crypts of the small intestine and red circle showing apoptotic bodies). **C** H&E staining of the skin of the recipient mice at day 7 post-transplant (black arrows showing the dermis of the skin and red circle showing increase of inflammatory cells) **D** H&E staining of the liver of the recipient mice at day 21 post-transplant (black arrows showing the interlobular bile ducts and red circle showing inflammatory infiltrates). **E** H&E staining of the small intestines of the recipient mice at day 21 post-transplant (blacks arrows showing apoptotic bodies). **F** H&E staining of the skin of the recipient mice at day 21 (black arrows showing dermis of skin and red circle showing increased inflammatory cells with destructed adnexal glands)

During GVHD, host tissues are damaged by the activity of alloactivated T cells. To determine whether damage to target organs of GVHD (skin, liver, and small intestine) was altered by loss of TCF-1 in donor T cells, we collected organs from mice allotransplanted as described above [28, 35, 83]. At day 7 and day 21 post-transplant, we collected pieces of skin, small intestine, and liver from recipient BALB/c mice. These organs were fixed, sectioned, stained with hematoxylin and eosin (H&E), and analyzed by a pathologist (L.S.) (Fig. 5). At day 7, TCF-1 cKO mice showed significantly less inflammatory infiltrates in all the organs. We observed much less inflammatory infiltrates in the bile duct epithelium of the portal triad (black arrows showing the interlobular bile ducts) in the liver of the TCF-1 cKO-transplanted recipients compared with WT-transplanted recipients (Fig. 5A). In the small intestines, no apoptotic bodies were seen in the crypts of the small intestine in the TCF-1 cKO CD8 T cell-transplanted mice, while frequent apoptotic bodies were present in the WT CD8 T cell-transplanted mice at day 7 post-transplantation (black arrows) (Fig. 5B). In the skin, a mild increase in inflammatory cells was observed in the dermis of the WT CD8 T transplanted mice, while the dermis of the TCF-1 cKO CD8 T transplanted mice appears normal at day 7 post-transplantation (Fig. 5C).

Again, at day 21 post-transplant, TCF-1 cKO CD8 T cell-transplanted mice showed significant less inflammatory infiltrates in all the sectioned GVHD target organs. We observed much less inflammatory infiltrates involving the bile duct epithelium of the portal triad (black arrows showing the interlobular bile ducts) in the liver of the TCF-1 cKO CD8 T cell-transplanted mice compared with WT CD8 T cell-transplanted mice (Fig. 5D). At day 21 post-transplant, no apoptotic bodies were seen in the crypts of the small intestines of the TCF-1 cKO CD8 T cell-transplanted mice, while few apoptotic bodies are present in the small intestines of the WT CD8 T cell-transplanted mice (black arrows) (Fig. 5E). A marked increase in inflammatory cells (red circle) and destruction of the adnexal glands was observed in the dermis of the WT CD8 T cell-transplanted recipients, while the dermis of the TCF-1 cKO CD8 T cell-transplanted recipients showed only a mild increase in dermal inflammatory cells, with preservation of adnexal glands (Fig. 5F). Altogether, these data suggest that TCF-1 normally promotes GVHD damage to healthy tissues and is indispensable for T cell-driven damage. Thus, loss of TCF-1 in donor T cells leads to reduced severity and persistence of GVHD over time.

### TCF-1 alters the transcriptomic signature of alloactivated T cells

Given that the phenotype and functions of donor T cells, as well as disease outcomes, were significantly altered by loss of *TCF-1* on donor cells, we sought to determine what specific gene changes occurred to support this. We allotransplanted recipient BALB/c mice with 1 × 10^6^ donor CD3 T cells as above, and FACS-sorted pre- and post-transplant WT or TCF-1 cKO donor CD8 T cells, which were stored in Trizol and transcriptionally profiled. When we analyzed the genetic profile of the pre-transplanted CD8 T cells from TCF-1 cKO and WT mice, we were not able to determine any differentially expressed genes (DEGs, FDR < 0.1). However, when we performed gene set enrichment analysis (GSEA) using the Hallmark pathways collection from Molecular Signatures Database (MSigDB), we identified that cytokine signaling pathways like TNF-*α* Signaling via NF-*κβ* and Interferon gamma response were enriched in pre-transplanted CD8 T cells from WT mice compared with cells from TCF-1 cKO mice (Supp. Fig. 5A-C). Meanwhile, a number of pathways involved in cell cycle also were enriched in WT cells versus TCF-1 cKO pre-transplanted CD8 T cells, like the P53 pathway, G2M checkpoint, DNA repair, and Myc targets pathways (Supp. Fig. 5A). MTOR signaling, allograft rejection, and oxidative phosphorylation pathways were also enriched in pre-transplant CD8 T cells from WT mice (Supp. Fig. 5A,6D-F), suggesting that loss of TCF-1 alters the transcriptional profile of CD8 T cell toward decreased cytokine release while also altering the cell cycle, leading to a lessening of the alloactivation responses.

**Fig.6:**
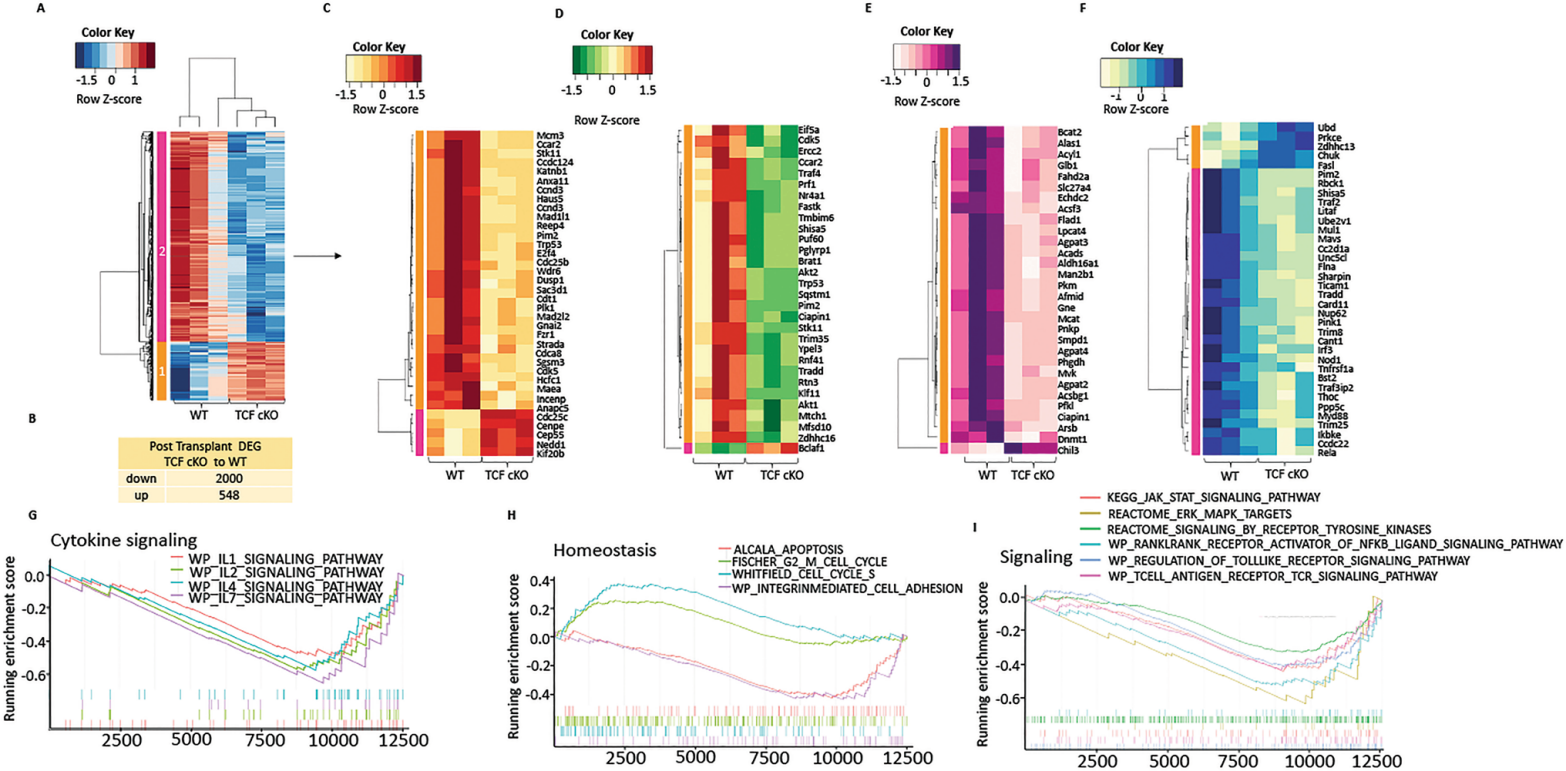
Loss of TCF-1 changes the genetic signature of alloactivated donor CD8 T cells. CD8 T cells from WT and TCF-1 cKO mice were FACS-sorted either into Trizol as pre-transplanted samples, or into 10% FBS-containing media for transplantation into recipient mice as described previously. At day 7 post-transplant, donor CD8 T cells were FACS-sorted back from recipient spleens of TCF-1 cKO- and WT-transplanted mice. RNA was extracted and prepped by the Molecular Analysis Core (SUNY Upstate). Paired end sequencing was done with an Illumina NovaSeq 6000 system at the University at Buffalo Genomics Core. For data analysis, we used the statistical computing environment R (v4.0.4), the Bioconductor suite of packages for R and RStudio (v1.4.1106). We calculated the transcript abundance by performing pseudoalignment using Kallisto. **A** Hierarchical clustering of genes and samples, heatmap illustrating the expression of the differentially expressing genes (DEG’s; FDR < 0.1) of post-transplanted CD8 T cells TCF-1 cKO compared to WT. **B** Table showing the number of up- or downregulated DEGs in post-transplanted CD8 T cells (TCF-1 cKO compared to WT). **C** Heatmap showing top significant 35 differentially expressed genes that play a role in the Cell Cycle pathway, identified using DAVID functional annotation analysis and GO-BP terms in post-transplanted CD8 T cells (TCF-1 cKO compared to WT)**. D** Heatmap showing top significant 30 differentially expressed genes that play a role in the apoptotic processes pathway, identified using DAVID functional annotation analysis and GO-BP terms in post-transplanted CD8 T cells (TCF-1 cKO compared to WT). **E** Heatmap showing top significant 30 differentially expressed genes that play a role in the metabolic processes pathway, identified using DAVID functional annotation analysis and GO-BP terms in post-transplanted CD8 T cells (TCF-1 cKO compared to WT). **F** Heatmap showing top significant 35 differentially expressed genes that play a role in the NF-*κβ* pathway, identified using DAVID functional annotation analysis and GO-BP terms in post-transplanted CD8 T cells (TCF-1 cKO compared to WT). **G** Gene set enrichment analysis (GSEA) enrichment plots of Cytokine signaling pathways, including IL-1, IL-2, IL-4, and IL-7 signaling pathways from WP terms that are enriched in post-transplanted CD8 T cells from WT mice (versus TCF-1 cKO). Negative enrichment score is an indicator of downregulation, and positive enrichment score is an indicator of upregulation of the genes in the post-transplanted CD8 T cells from TCF-1 cKo mice. DAVID enrichment scores > 1.3 are equivalent to a *P* value < 0.05 **H** GSEA enrichment plots of G2 to M cell cycle and cell cycle S pathways that are enriched in TCF-1 cKO mice, and integrin-mediated cell adhesion and apoptosis pathways that are enriched in post-transplanted CD8 T cells from WT mice. **I** GSEA enrichment plots of JAK-STAT signaling, toll-like receptor signaling, T cell receptor signaling, ERK-MAPK signaling, signaling by tyrosine kinases, RANKL—RANK-mediated NF-*κβ* signaling pathways that are enriched in post-transplanted CD8 T cells from WT mice. Again, negative enrichment score is an indicator of downregulation, and positive enrichment score is an indicator of upregulation of the genes in the post-transplanted CD8+ T cells from TCF-1 cKo mice. DAVID enrichment scores > 1.3 are equivalent to a *P* value < 0.05

When we analyzed the post-transplanted CD8 T cells which were alloactivated in vivo, we identified 2548 differentially expressed genes (DEGs; FDR < 0.1) when comparing TCF-1 cKO cells to WT cells (Fig. 6A–B). A majority of the DEGs (2000 genes) were downregulated (module 2 in heatmap) and only 548 genes (module 1 in heatmap) were upregulated in post-transplant CD8 T cells from TCF-1 cKO mice compared to WT mice (Fig. 6A–B). We analyzed both up- and downregulated DEGs for the gene ontology (GO) enrichment analysis using functional annotation chart tools, selecting only the top 20 GO-BP (Biological Process) terms in the Database for Visualization and Integrative Discovery (DAVID) [89, 90]. The analysis showed that the differentially expressed genes in post-transplant CD8 T cells from TCF-1 cKO mice compared to WT were involved in cell cycle, cell–cell adhesion, cell division, apoptotic process, antigen processing and presentation via MHC class I, TCR signaling, regulation of NF-*κβ* signaling, metabolic process, and others (Supp. Fig. 6). Once we knew which processes these DEGs played a role in, we pulled out the top genes that were altered for each GO-BP term that we were interested in. When we looked at the top 35 DEGs based on *P* value that were altered in cell cycle, we observed that a majority of them were downregulated in TCF-1 cKO compared to WT (Fig. 6C). We also looked at the top 30 genes based on *P* value that were altered in Apoptotic processes (Fig. 6D), which also showed that most of the genes were downregulated in CD8 T cells in TCF-1 cKO mice. When we looked at the top 30 genes that play a role in metabolic processes, we observed that only 1 gene was upregulated, and the rest were downregulated in in vivo alloactivated CD8 T cells from TCF-1 cKO mice (Fig. 6E). While the upregulated genes in the NF-*κβ* pathway were Fasl, Ubd, Chuk, and others, the downregulated genes were Rela, Irf3, Traf2, Mavs, Tradd, Nod1, Tnfrsf1a, and Trim25 (Fig. 6F).

Gene set enrichment analysis (GSEA) using the Hallmark pathways identified that signaling pathways like PI3K-AKT-MTOR signaling and TNFA signaling via NF-*κβ* were enriched in post-transplanted CD8 T cell from WT mice compared to cells from TCF-1 cKO mice (Supp. Fig. 7A-B). GSEA analysis using the C2 canonical pathways showed that a number of cytokines signaling pathways involving IL-1, IL-2, IL-4, and IL-7 were enriched in post-transplanted CD8 T cells from WT mice compared to cells from TCF-1 cKO mice (Fig. 6G). While cell cycle pathways were enriched in CD8 T cells from TCF-1 cKO mice compared to cells from WT mice, apoptosis and cell adhesion pathways were enriched in post-transplanted CD8 T cells from WT mice (Fig. 6H). Meanwhile, a number of cells signaling pathways were also enriched in WT CD8 T cells compared to TCF-1 cKO cells, such as TCR signaling, toll-like receptor signaling, Jak-Stat signaling, ERK-MAPK signaling, and NF-*κβ* pathways (Fig. 6I).

**Fig.7:**
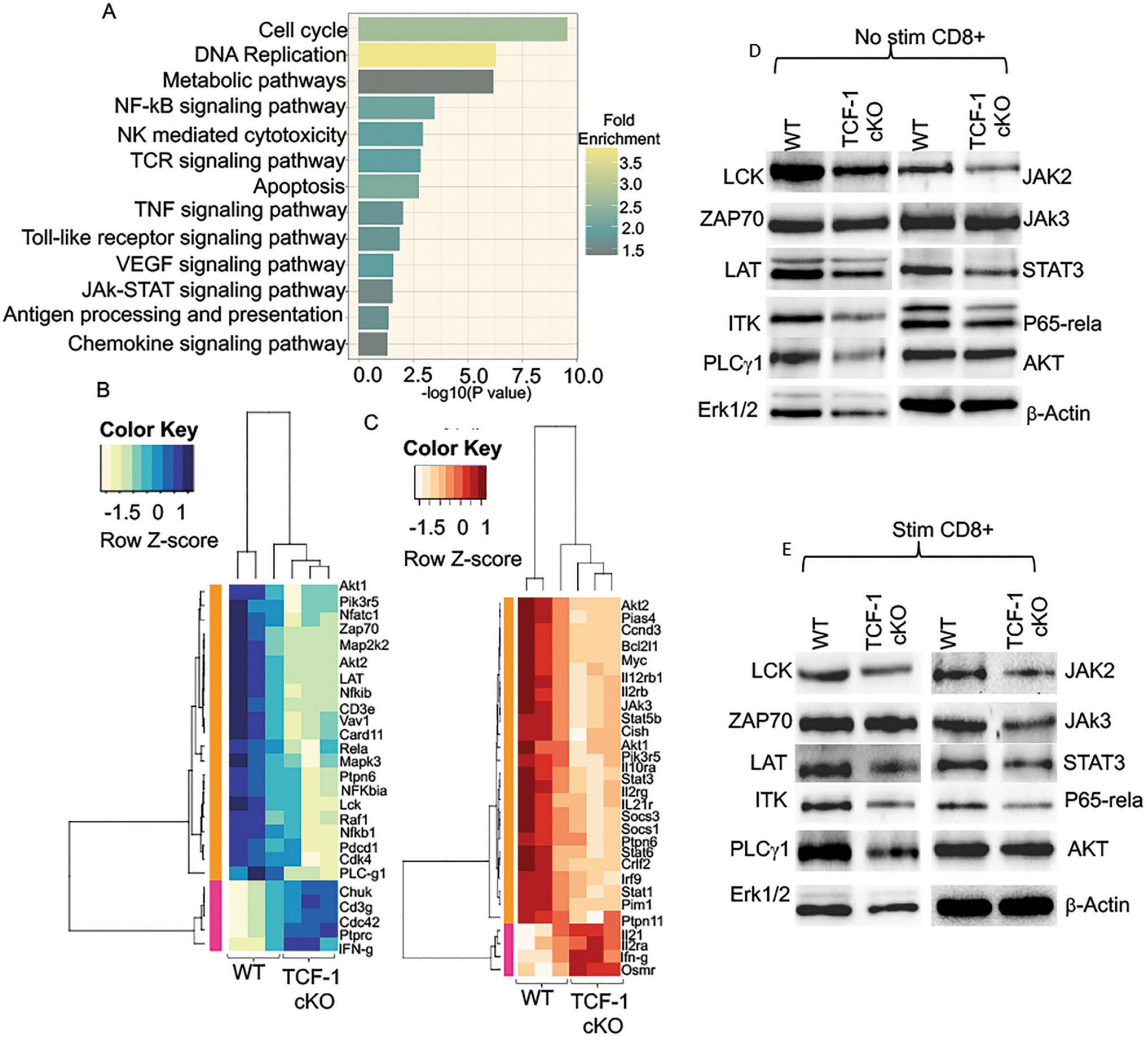
Loss of TCF-1 decrease TCR, JAK-STAT, and NF-*κβ* signaling downstream. **A** A bar plot showing KEGG (Kyoto Encyclopedia of Genes and Genomes) pathways identified by DAVID functional annotation analysis. **B** Heatmap showing the altered genes in TCR signaling pathway from KEGG pathways. **C** Heatmap showing the altered genes in JAK-STAT signaling pathway from KEGG pathways. **D** Western blot showing the protein expression levels of LCK, ZAP70, LAT, ITK, PLC*γ*1, ERK1/2, JAK2, JAK3, STAT3, P65-RelA, AKT, and β-actin of freshly isolated CD8 T cell lysates from TCF-1 cKO and WT mice. **E** Western blot showing the protein expression levels of LCK, ZAP70, LAT, ITK, PLC*γ*1, ERK1/2, JAK2, JAK3, STAT3, P65-RelA, AKT, and β-actin in 10 min-CD3/CD28-stimulated CD8 T cell lysates from TCF-1 cKO and WT mice. All the Western blots repeated at least three and one representative of each protein and quantification is shown

We also analyzed the genes that were altered in KEGG pathways, which revealed that DEGs that were altered in in vivo alloactivated CD8 T cells from TCF-1 cKO mice (compared to WT) were involved in pathways like cell cycle, DNA replication, metabolic pathways, natural killer-mediated cytotoxicity, TCR signaling, JAK-STAT signaling, chemokine receptor signaling, and others (Fig. 7A). Specifically, Klrk1 gene for NKG2D on natural killer-mediated cytotoxicity pathway was enriched in alloactivated TCF-1 deficient CD8+ T cells compared to WT CD8 + T cells. Clustering of genes that were affected in TCR signaling showed that while AKT1, AKT2, Pik3r5, Zap70, LCK, Lat, PLC*γ*1, Pdcd1, Vav1, Rela, Mapk3, Nfkbia, and Nfatc1 were downregulated, Ifng and Ptprc were upregulated in post-transplanted CD8 T cells from TCF-1 cKO mice (Fig. 7B). The JAK/STAT signaling pathway is important for cytokine production and for the response of T cells to cytokines. Analysis revealed that IL2RB, JAK3, STAT5B, STAT3, STAT1, Cish, Il2rb, Socs3, and Socs1 genes were downregulated in the JAK-STAT pathway for TCF-1 cKO CD8 T cells compared to WT (Fig. 7C).

In order to confirm these changes in genes, we isolated CD8 T cells from WT and TCF-1 *cKO* mice and determined the baseline level protein expression of LCK, ZAP70, LAT, ITK, PLC*γ*1, ERK1-2, Jak2, Jak3, Stat3, p65-rela, AKT, and actin in unstimulated and 10 min-anti-CD3/CD28-stimulated samples. Data from non-stimulated samples revealed that protein expression of most of the markers was downregulated in CD8 T cells from TCF-1 cKO mice compared to WT mice; only ZAP70, JAK3, and AKT were not affected by loss of *TCF-1* (Fig. 7D, Supp. Fig. 8A). We observed even more robust differences in samples that were stimulated with anti-CD3/CD28 for 10 min, and again only ZAP70 and AKT were unaffected by loss of TCF-1 (Fig. 7E, Supp. Fig. 8B). Altogether, the data from RNA sequencing analysis and Western blots of stimulated and unstimulated samples showed attenuation of TCR signaling and many other pathways in CD8 T cells from TCF-1 cKO mice. These results help to explain why CD8 T cells from TCF-1 cKO mice cannot induce GVHD as severely as CD8 T cells from WT mice.

## Discussion

T Cell Factor-1 (TCF-1) is a critical regulatory transcription factor in T cell development and functions [91]. TCF-1 is known to be important for T cell development, as well as activation in some contexts [45]. TCF-1 has been extensively studied in viral infection [1, 10–12, 92, 93]. However, the role of (TCF-1) in a mouse model of allogeneic transplant has not been investigated. Using a murine allogeneic transplant model, we have shown that CD8 T cells from TCF-1 cKO effectively clear tumor cells without inducing GVHD by producing significantly less inflammatory cytokines [31], and cause significantly less tissue damage in GVHD target organs [94, 95]. Here, we show that loss of TCF-1 alters a number of CD8 T cell functions, and while it is dispensable for anti-tumor responses, it is essential for host tissue damage, cytokine production and signaling, and gene expression, playing a role in a number of immunological and biological pathways during alloactivation [45]. These studies provide evidence for how TCF-1 regulates the functions of peripheral T cells. The phenotype caused by loss of TCF-1 is clinically optimal, because it allows for clearance of residual malignant cells while limiting the risk of life-threatening GVHD damage [96]. This observation, coupled with the increase in exhaustion of TCF-1 cKO donor CD8 T cells, suggests that donor cells lacking TCF-1 are highly activated and cytotoxic to malignant cells early on following transplant, but quickly become exhausted, limiting GVHD progression.

TCF-1, Eomes, and T-bet play central roles in orchestrating T cell response to tumors or pathogens in individuals [61, 68, 97–99]. Whether TCF-1 suppresses Eomes and T-bet expression was not known previously. Thus, the main significance of our results was showing that TCF-1 suppresses Eomes and T-bet and negatively regulates Granzyme B expression. These findings correlate with CD122 and CD44 expression, which are key CD8 T cell activating markers [28, 30, 35]. T cell effector memory (EM), central memory (CM), and naïve subsets have been shown to determine T cell functional characteristics [100, 101]. We have shown that CD8 T cells from TCF-1 cKO mice showed increases in both CM and EM and decreases in naïve CD8 T cells [30]. These findings correlate with the higher expression of Eomes and T-bet. We and others have shown that CD8 T cells with increased EM and CM do not cause GVHD [28, 30, 33–35, 102].

CD8 T cells utilize CD28 as a major co-stimulatory molecule for effector function [103]. CD28 not only functions to magnify TCR signaling, but also controls key biochemical events important for post-translational protein modification (e.g., phosphorylation) and epigenetic changes that alter the gene expression program of T cells [104]. CD8 T cells also utilize NKG2D as a co-stimulatory molecule [39, 77]. However, their independent functional contributions in distinct CD8+ T cell subsets are not well understood [105]. Published data have also shown that mouse naïve CD8 T cells express higher levels of NKG2D when CD28 expression is decreased [106]. However, the mechanism of what regulates these process were not known previously. Our data uncovered that CD8 T cells from TCF-1 cKO mice abolished CD28 expression and utilized NKG2D expression as a major co-stimulatory receptor. Our data provided compelling evidence that TCF-1 suppresses NKG2D expression and inhibits NKG2D mediated cytolytic function. These data provided evidence that TCF-1 is critical for CD8 T cells stemness but might be dispensable for anti-tumor response. Our findings will provide platform for modulating key signaling can uncouple GVHD from GVL.

TCF-1 regulates several key pathways including cell cycle, DNA repair, metabolic pathways, NF-*κβ* pathways, apoptosis, TNF, signaling, tool-like receptor signaling, VEGF signaling, and many other signaling pathways. In this report, we specifically focused on TCR signaling and cytokine production pathways to provide evidence that TCF-1 is required for TCR stemness, but TCF-1 is dispensable for CD 8 T cell cytolytic function.

The current limitations of our studies are that we used genetic approaches, and modulating molecules with genetic approaches in the clinic is still challenging. Our data agree with previous studies providing strong evidence that strategies can be developed to overcome these limitations. One possible approach could be utilizing glycogen synthase kinase-3 (GSK3), which is a ubiquitously expressed, highly conserved serine/threonine protein kinase found in all T cells [107]. GSK3 inhibitors such as Tideglusib, LY2090314, Enzastaurin, and LiCl [108] can be used to inhibit TCF-1 expression.

Altogether, these data suggest that TCF-1 is a major transcription factor that plays a role in T cell development. Our work shows that TCF-1 is dispensable for cytotoxic function of mature alloactivated CD8 T cells but is indispensable for GVHD. TCR, JAK-STAT, and NF-*κβ* signaling as well as cytokine production, these findings will help to establish an understanding of TCF-1 as a critical factor in the GVHD/GVL regulatory network of CD8 T cells.

## Supplementary Information

Below is the link to the electronic supplementary material.

Supplementary file1 (DOCX 1708 KB)
