# Supplementary material for: TCF-1 regulates NKG2D expression on CD8 T cells during anti-tumor responses
Source: Cancer Immunol Immunother. 2022 Dec 23;72(6):1581–601. doi: 10.1007/s00262-022-03323-0 (PMC10198945; doi:10.1007/s00262-022-03323-0)
Supplement: Supplementary file 1 — Supplementary file1 (DOCX 1708 KB) [file 262_2022_3323_MOESM1_ESM.docx]

**Supplemental Figure Legends:**

**
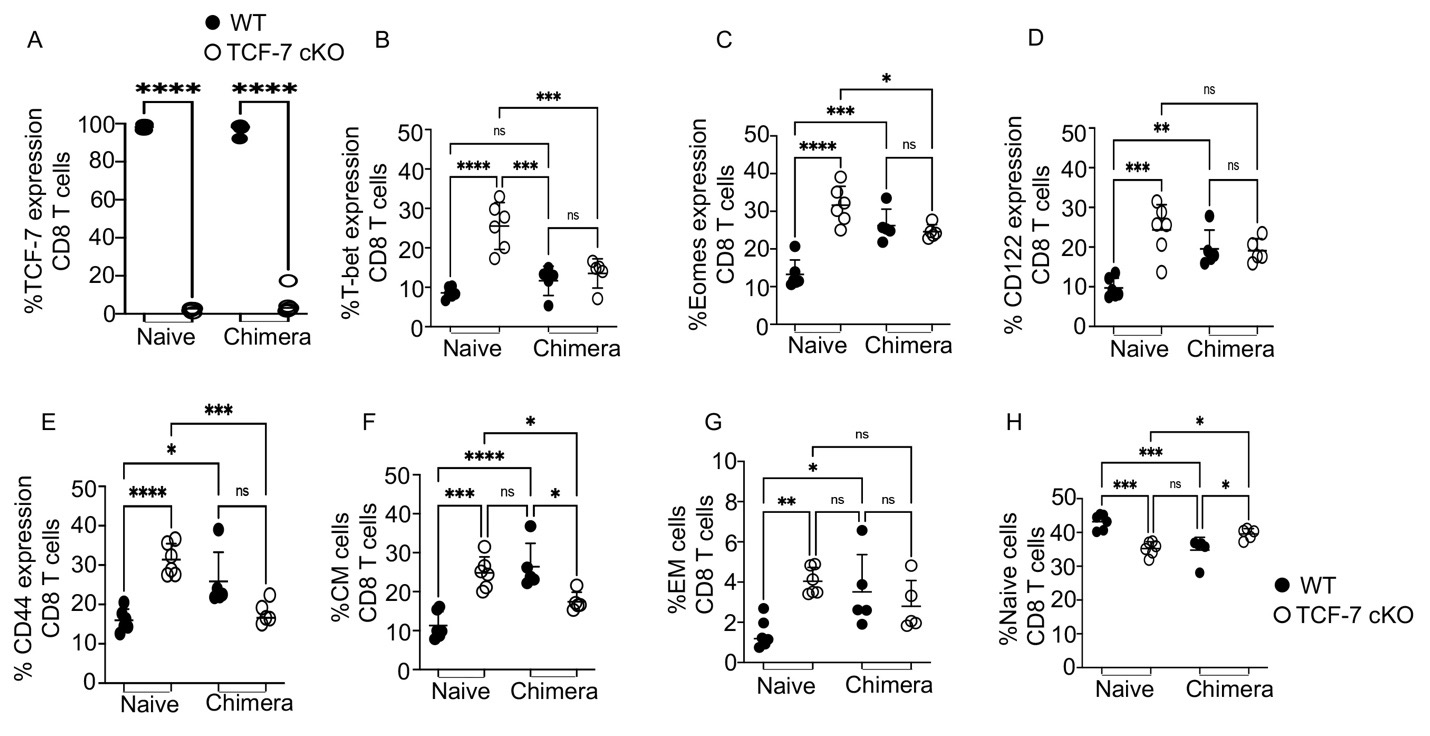
**

**Supp.Fig.1. Related to Fig2. Loss of *TCF-7* drives changes to mature CD8 T cell phenotype that are cell-intrinsic, with the possibility of extrinsic effects.** Bone marrow chimeras were developed by lethally irradiating Thy1.1 mice and reconstituting with a 1:4 (WT:*TCF-7* cKO) mixture of bone marrow cells. Blood was tested at 9 weeks to ensure reconstitution with both donor cell types, and splenocytes were used at 10 weeks for phenotyping by flow cytometry. **(A)** Percentage of CD8 T cells from chimeric and naive mice expressing *TCF-7*. **(B)** Percentage of CD8 T cells from chimeric and naive mice expressing T-bet. **(C)** Percentage of CD8 T cells from chimeric and naive mice expressing Eomes. **(D)** Percentage of CD8 T cells from chimeric and naive mice expressing CD122. **(E)** Percentage of CD8 T cells from chimeric and naive mice expressing CD44. **(F)** Percentage of CD8 T cells from chimeric and naive mice expressing central memory (CM) phenotype. **(G)** Percentage of CD8 T cells from chimeric and naive mice expressing effector memory (EM) phenotype. **(H)** Percentage of CD8 T cells from chimeric and naive mice expressing naïve phenotype. All data are plotted as individual points with mean and SD, all were analyzed with one-way ANOVA, or Student’s t-test (depending on groups). For all graphs, * means p-value ≤ 0.05, *** means p-value ≤ 0.001, and **** means p-value ≤ 0.0001. For naïve cells 3 different experiments combined (N=2-3 per group of mice) and for chimera cell (N=5) with one
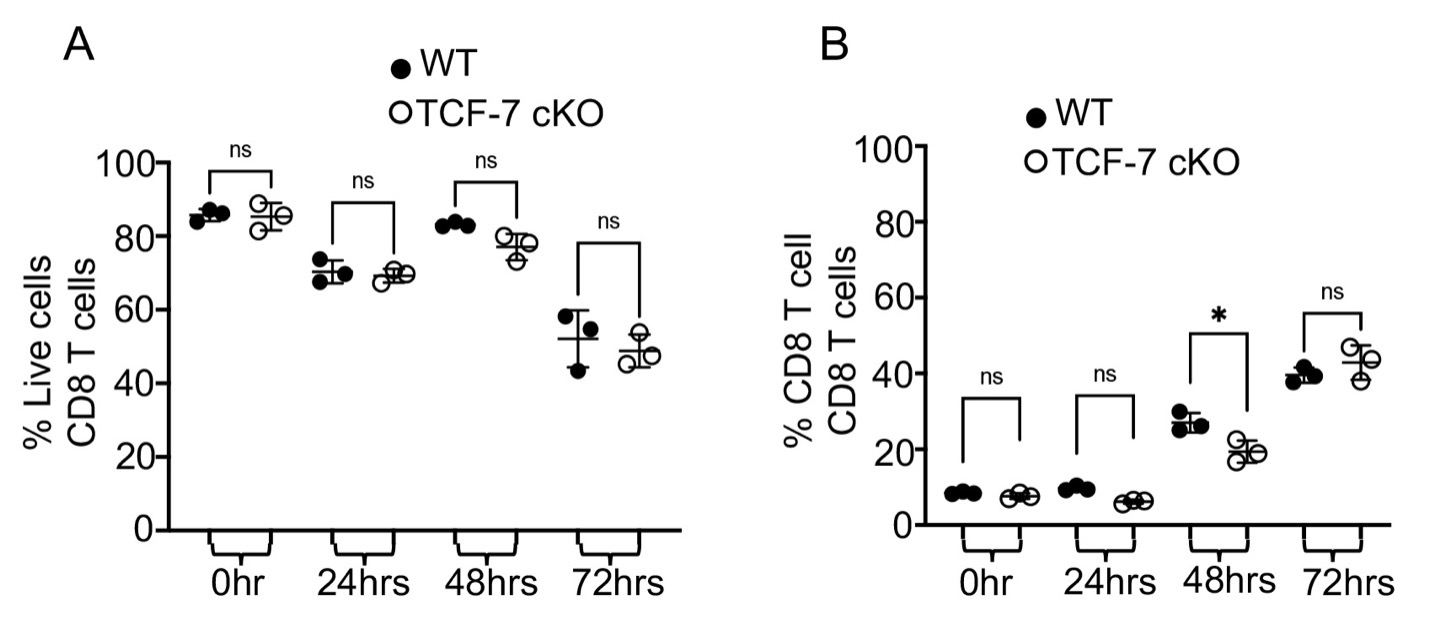
experiment shown (done once).

**Supp.Fig.2 Related to Fig.3. Cell viability and CD8 + T cell percent in NKG2D induction *in vitro*.** Total splenocytes were isolated from *TCF-7* cKO or WT mice and either left unstimulated or stimulated with anti-CD3/CD28 for 24, 48, or 72 hrs in culture. GolgiPlug (1:1000) was added to stimulated samples for each time point except 0 hr samples, and samples were incubated at 37 C with 7% CO2. After 6 hours of culture, the cells were stained for CD3, CD8, NKG2D, and Granzyme B expression, as determined by flow cytometry**. (A)** Quantification of percent of live cells (dead cells that were positive for LIVE/DEAD Aqua excluded) for different time points of stimulation. **(B)** Quantification of CD8 T cell percentages for different time points of stimulation. N=4 per group with one representative of 2 experiments shown. All data are shown as individual points with mean and SD, and were analyzed with two-way ANOVA (depending on groups). * means p-value ≤ 0.05, ** means p-value ≤ 0.01, and *** means p-value ≤ 0.001.

**
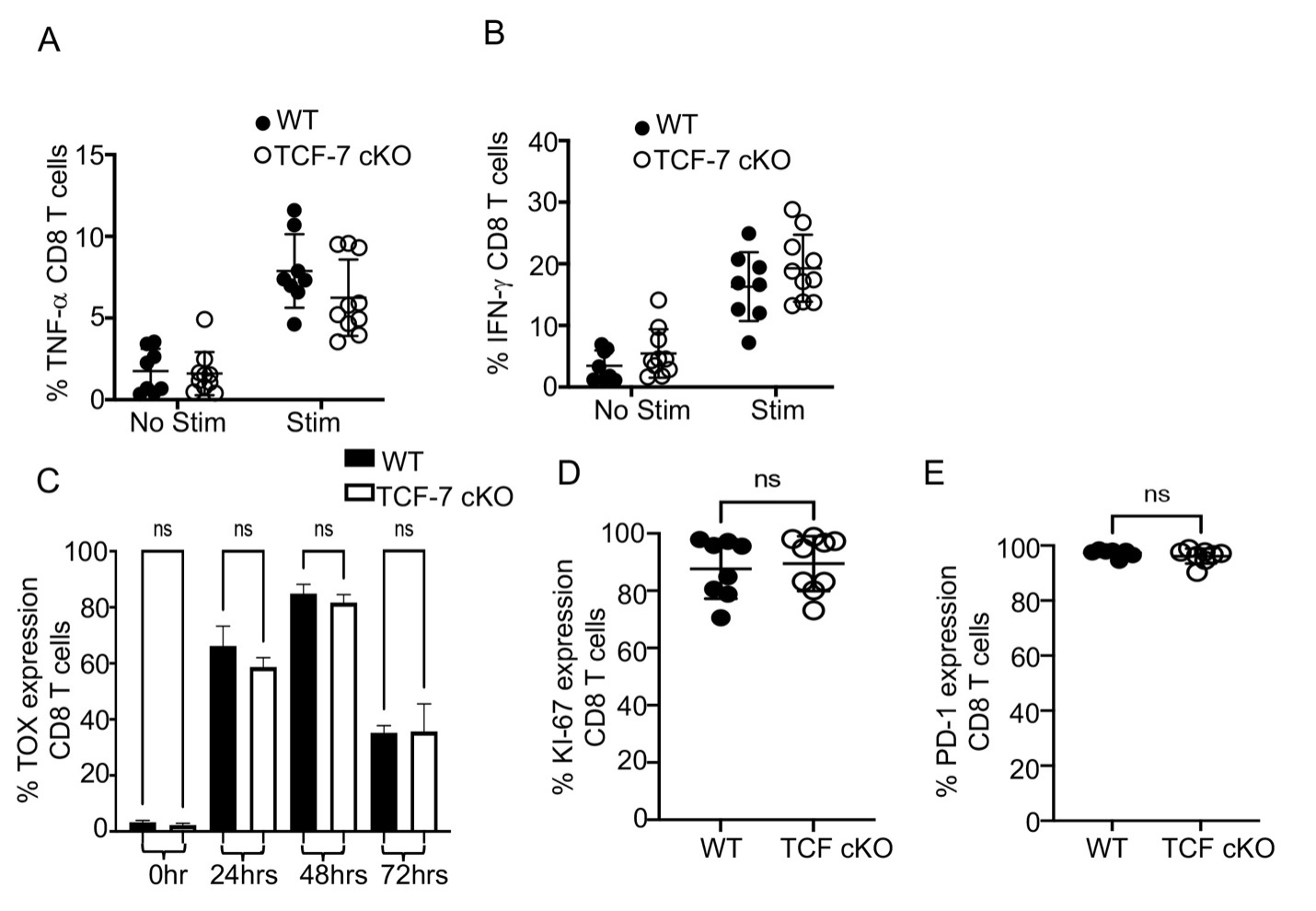
**

**Supp.Fig3. Related to Fig.5. *TCF-7* controls cytokine production and exhaustion of mature alloactivated CD8 T cells. (A-B)** Recipient mice were allotransplanted with 1.5X10^6^ WT or *TCF-7* cKO donor CD3 T cells as before. Splenocytes were taken at day 7 post-transplant, restimulated by 6-hour culture with Golgi Plug and PBS (control) or anti-CD3/anti-CD28 (TCR restim.), then stained for H2K^b^, CD3, CD4, CD8, TNF-α, and IFN-γ. **(A)** TNF-α production and **(B)** IFN-γ production by donor CD8 T cells, as measured by percent cytokine-positive donor cells. **(C)** Splenocytes from *TCF-7* cKO and WT mice were obtained and either stimulated with anti-CD3/CD28 antibodies for 24hrs, 48hrs, or 72hrs in culture and stained for TOX, or were immediately stained after isolation without stimulation. Percent expression of TOX in CD8 T cells after 72 hours of CD3/CD28 stimulation in culture, as determined by flow cytometry. **(D-E)** Balb/c mice were allo-transplanted as before, with WT BM and WT or *TCF-7* cKO CD8 donor T cells. On day 7, spleens were removed from recipients, processed to isolate lymphocytes, and lymphocytes were stained for CD3, CD8, H2K^b^, Ki-67, and PD-1 to identify proliferating and exhausted T cells, respectively. **(D)** Quantification of the Ki-67 expression in *in vivo* donor CD8 T cells. (**E)** Quantification of the PD-1 expression in *in vivo* donor CD8 T cells. N=3-5 per group for **A-B and D-E** with two experiments shown. N=4 per group for **C,** one representative of two experiments shown. All data are shown as individual points with mean and SD, and were analyzed with Student’s t-test or two-way ANOVA (depending on groups). * means p-value ≤ 0.05
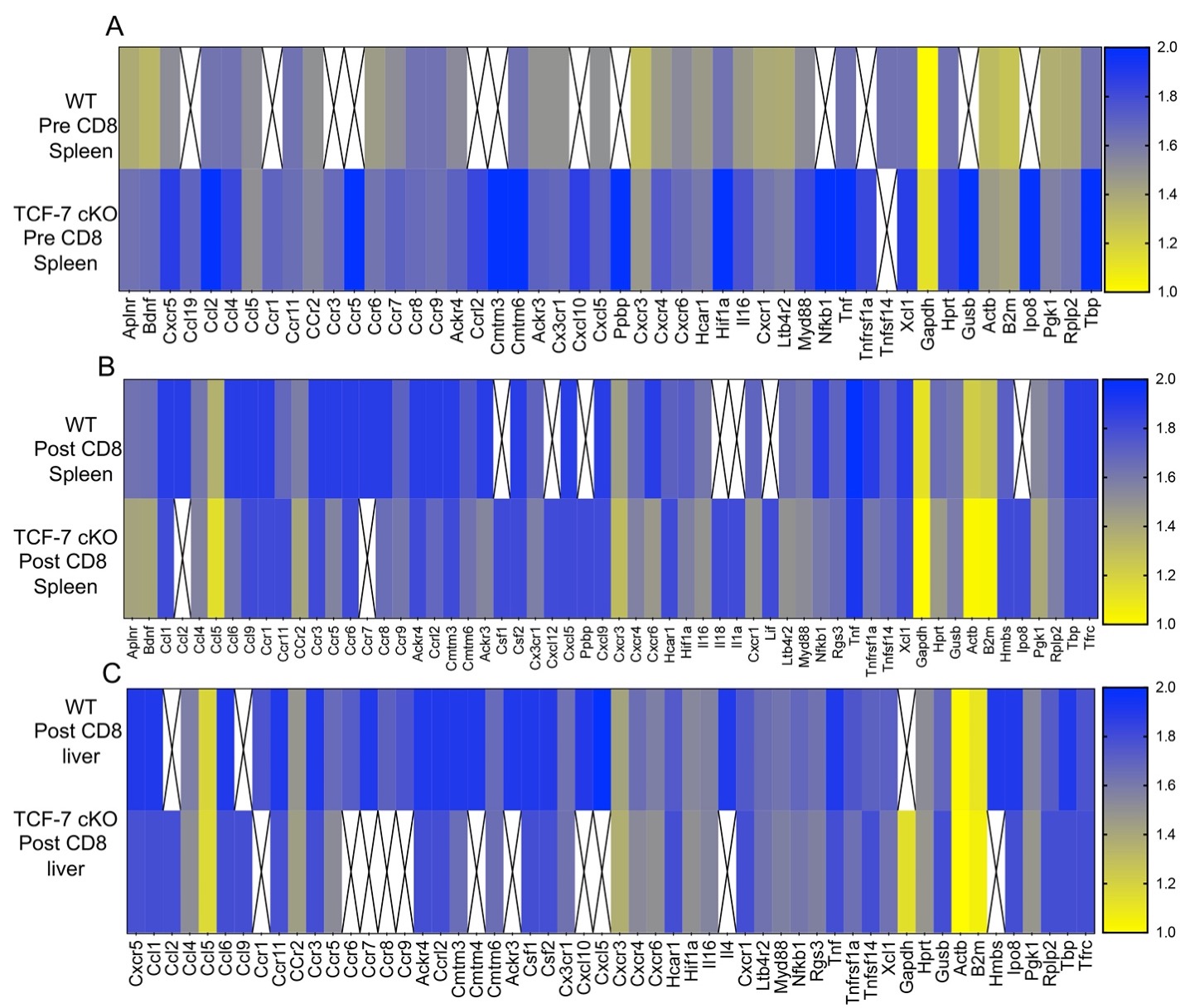
, ** means p-value ≤ 0.01, and *** means p-value ≤ 0.001.

**Supp.Fig.4. *TCF-7* controls chemokine and chemokine receptor expression of donor CD8 T cells during alloactivation.** As before, BALB/c mice were allotransplanted with BM and 1X10^6^ donor CD3 T cells from WT or *TCF-7* cKO mice (not mixed). Donor CD8 T cells were FACS-sorted from spleen of donor’s pre-transplant, and from spleen and liver of recipients at day 7 post-transplant. Cells were sorted into Trizol, then RNA was extracted using chloroform and converted to cDNA for qPCR analysis. cDNA was run on premade mouse chemokine/chemokine receptor assay plates, and results are displayed as heatmaps. Scales are shown at right, with fold change per gene compared to an 18S reference gene on each plate. White boxes with an “X” represent signals too low to detect or otherwise unreadable due to technical limitation/error. **(A)** Pre-transplant spleen donor CD8 T cells, **(B)** post-transplant spleen donor CD8 T cells, and **(C)** post-transplant liver donor CD8 T cells for WT versus *TCF-7* cKO donors. N=5 mice into one sample per condition**,** summary data shown.

**
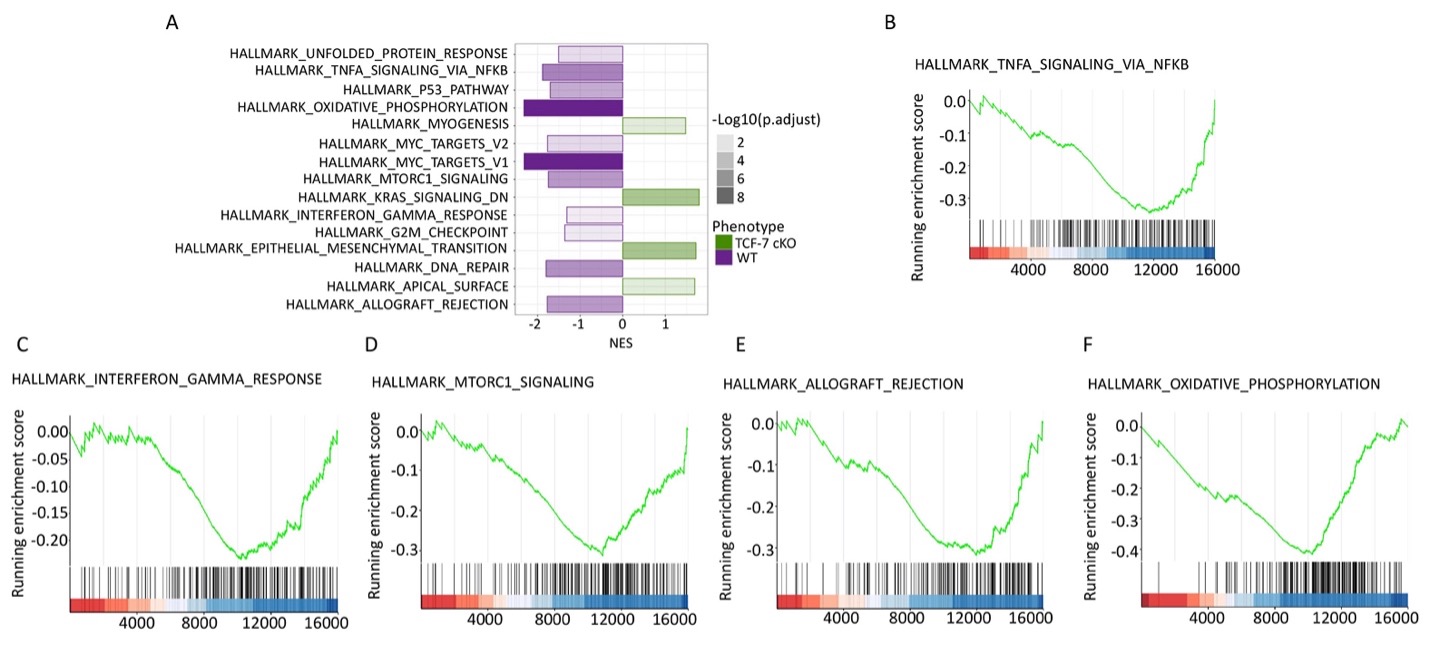
**

**Supp.Fig.5: Loss of *TCF-7* alters the enrichment of gene sets in pre-transplanted CD8+T cells. (A)** Bar plot of the altered pathways indentified in Gene set enrichment analysis (GSEA) by using Hallmark gene set collections of the Molecular Signatures Database (MSigDB) in pre-transplanted CD8 T cells (*TCF-7* cKO compared to WT). The normalized enrichment score (NES) for the pathway is defined as the peak score furthest from zero, with a negative NES meaning enrichment in the WT group.  **(B)** GSEA plot for the “HALLMARK_TNFA_SIGNALING_VIA_NFKB” pathway comparing pre-transplanted CD8 T cells from TCF-7 cKO to WT cells*.* The running enrichment score (ES) for the pathway is defined as the peak score furthest from zero, with a negative ES meaning enrichment in the WT group. **(C)** GSEA plot for the “HALLMARK_INTERFERON_GAMMA_RESPONSE” pathway comparing pre-transplanted CD8 T cells from TCF-7 cKO to WT cells*.* **(D)** GSEA plot for the “HALLMARK_MTORC1_SIGNALING” pathway comparing pre-transplanted CD8 T cells from TCF-7 cKO to WT cells*.* **(E)** GSEA plot for the “HALLMARK_ALLOGRAFT_REJECTION” pathway comparing pre-transplanted CD8 T cells from TCF-7 cKO to WT cells*.* **(F)** GSEA plot for the “HALLMARK_OXIDATIVE_PHOSPHORYLATION” pathway comparing pre-transplanted CD8 T cells from TCF-7 cKO to WT cells*.*

**
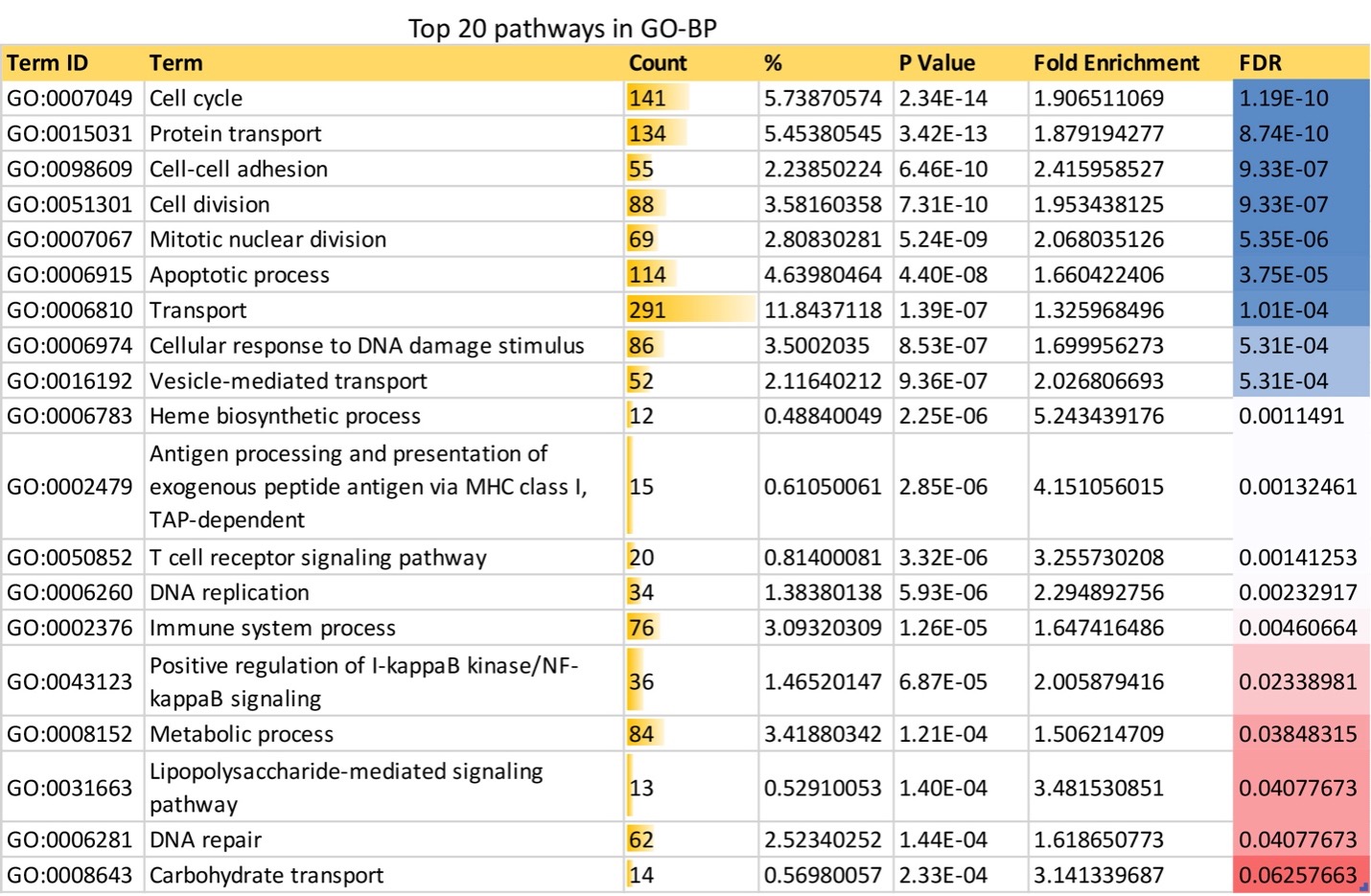
**

**Supp.Fig.6. Related to Fig.7**. Top 20 GO-BP terms identified in DAVID functional annotation analysis of differentially expressed genes in post-transplanted samples. Count mean – number of genes involved in the Term, % - percentage of involved genes/ total genes in the Term.

**
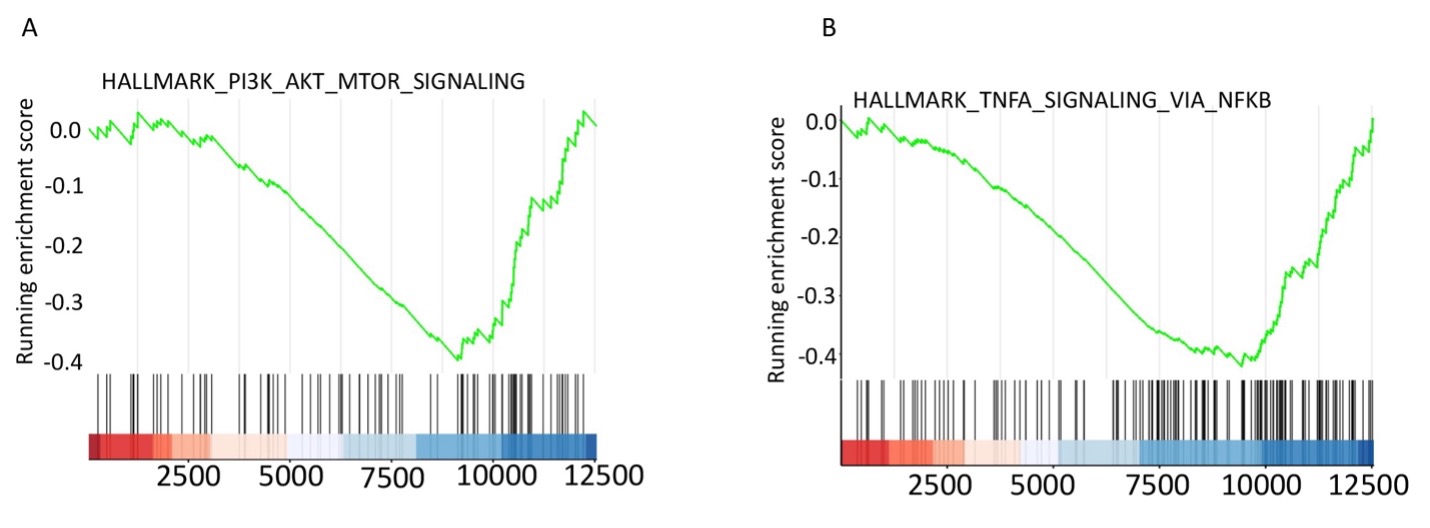
**

**Supp.Fig.7.** **Loss of *TCF-7* alters the Gene Set Enrichment Analysis (GSEA) of post-transplanted CD8+T cells. (A)** GSEA plot for the “HALLMARK_PI3K_AKT_MTOR_SIGNALING” pathway comparing post-transplanted CD8 T cells from TCF-7 cKO to WT mice*.* The running enrichment score (ES) for the pathway is defined as the peak score furthest from zero, with a negative ES meaning enrichment in the WT group. **(B)** GSEA plot for the “HALLMARK_TNFA_SIGNALING_VIA_NFKB” pathway comparing post-transplanted CD8 T cells from TCF-7 cKO to WT mice*.* Again, the running enrichment score (ES) for the pathway is defined as the peak score furthest from zero, with a negative ES meaning enrichment in the WT group.

**
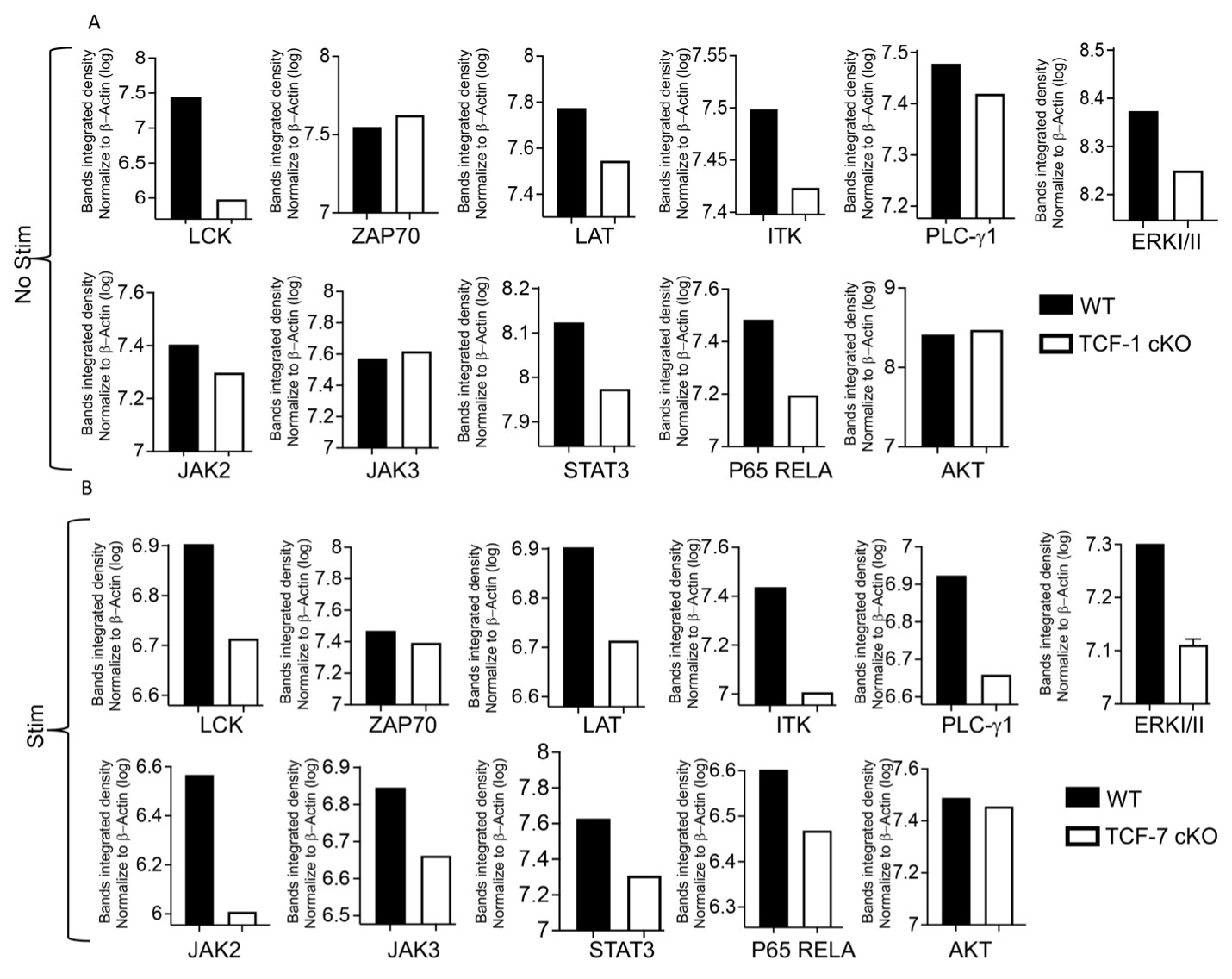
**

**Supp.Fig.8. Related to Fig.8**. **Quantification of Western blot of TCR and JAK-STAT signaling.** (**A)** Comparison of the quantified bands integrated density normalized to β-actin for unstimulated CD8 T cells from *TCF-7* cKO and WT mice**. (B)** Comparison of the quantified bands integrated density normalized to β-actin for 10-minute-anti-CD3/CD28-stimulated CD8 T cells from *TCF-7* cKO and WT mice. All the western blots repeated at least three times and one representative of each protein and quantification is shown.
